# The Intensive Care Lifeboat: a survey of lay attitudes to rationing dilemmas in neonatal intensive care

**DOI:** 10.1186/s12910-016-0152-y

**Published:** 2016-11-08

**Authors:** C. Arora, J. Savulescu, H. Maslen, M. Selgelid, D. Wilkinson

**Affiliations:** 1 Faculty of Medicine, Nursing and Health Sciences, Monash University, Melbourne, VIC Australia; 2 Monash Hospital, Clayton, VIC Australia; 3 Oxford Uehiro Centre for Practical Ethics, University of Oxford, Suite 8, Littlegate House, St Ebbes St, Oxford, OX1 1PT UK; 4 School of Philosophical, Historical and International Studies, Monash University, Melbourne, VIC Australia; 5 John Radcliffe Hospital, Oxford, UK

**Keywords:** Resource allocation, Intensive care units, Neonatal, Medical ethics, Questionnaires, Infant, Newborn, Resuscitation

## Abstract

**Background:**

Resuscitation and treatment of critically ill newborn infants is associated with relatively high mortality, morbidity and cost. Guidelines relating to resuscitation have traditionally focused on the best interests of infants. There are, however, limited resources available in the neonatal intensive care unit (NICU), meaning that difficult decisions sometimes need to be made. This study explores the intuitions of lay people (non-health professionals) regarding resource allocation decisions in the NICU.

**Methods:**

The study design was a cross-sectional quantitative survey, consisting of 20 hypothetical rationing scenarios. There were 119 respondents who entered the questionnaire, and 109 who completed it. The respondents were adult US and Indian participants of the online crowdsourcing platform Mechanical Turk. Respondents were asked to decide which of two infants to treat in a situation of scarce resources. Demographic characteristics, personality traits and political views were recorded. Respondents were also asked to respond to a widely cited thought experiment involving rationing.

**Results:**

The majority of respondents, in all except one scenario, chose the utilitarian option of directing treatment to the infant with the higher chance of survival, higher life expectancy, less severe disability, and less expensive treatment. As discrepancy between outcomes decreased, however, there was a statistically significant increase in egalitarian responses and decrease in utilitarian responses in scenarios involving chance of survival (*P* = 0.001), life expectancy (*P* = 0.0001), and cost of treatment (*P* = 0.01). In the classic ‘lifeboat’ scenario, all but two respondents were utilitarian.

**Conclusions:**

This survey suggests that in situations of scarcity and equal clinical need, non-health professionals support rationing of life-saving treatment based on probability of survival, duration of survival, cost of treatment or quality of life. However, where the difference in prognosis or cost is very small, non-health professionals preferred to give infants an equal chance of receiving treatment.

**Electronic supplementary material:**

The online version of this article (doi:10.1186/s12910-016-0152-y) contains supplementary material, which is available to authorized users.

## Background

Advances in medical technology enable doctors to save the lives of newborn infants who would have previously died [1, 2]. Some of these infants have a low chance of survival with treatment, or may survive with significant morbidity and shortened life expectancy [3]. Guidelines relating to provision of intensive care for critically ill newborns have traditionally focused on the best interests of infants [4]. However, there are limited resources (including staff, equipment and physical space) available within neonatal intensive care units (NICU) [5], meaning that sometimes treatment that would be in the best interests of an infant is not available. In developed countries there may be ways of stretching existing resources, though these have impacts on the quality of care and outcome [6, 7]. Neonates may require transfer to other hospitals to receive treatment [5], with an associated increase in morbidity and mortality [8, 9]. In countries with limited intensive care facilities and transport options, infants who are unable to receive treatment die [10, 11]. Where multiple infants require treatment, difficult decisions need to be made about which infant to treat [12]. How should doctors allocate limited resources and decide which patients to treat?

In answering this question, moral philosophers have described ethical principles of distributive justice including utilitarianism, egalitarianism and prioritarianism. In the context of resource allocation, utilitarianism focuses on maximizing aggregate population health by directing treatment to patients with the best prognosis [13]. Cost-Effectiveness Analysis (CEA), QALYs and DALYs are operationalized versions of this [12]. Egalitarianism recognizes the importance of equal opportunity for equal need. It is the stated basis of the UK National Health Service [14]. Resource allocation approaches that apply the principle of egalitarianism include lottery and first-come, first-served [15]. Finally, prioritarianism involves giving priority to the worst-off, for example those who are already disadvantaged, or have the greater clinical need [16].

Another ethical way of thinking about justice in the contractarian tradition is to employ a “veil of ignorance” [17]. This involves choosing a principle or procedure while imagining you might be one of the potential people in need, without knowing your own position.

The majority of existing literature focuses on the ethical principles that ought to govern resource allocation decisions in the NICU, while empirical evidence provides insight into what factors actually govern the decision-making process as well as the views of stakeholders in the NICU. Most empirical studies in this area focus on the views of healthcare practitioners [18, 19], particularly neonatologists [19, 20], and parents [21, 22].

We sought to explore the views of lay people (non-health professionals) about resource allocation decisions in the NICU using a series of hypothetical rationing dilemmas designed to test egalitarian or utilitarian intuitions. The views of the general public do not resolve ethical questions, but are relevant in realising the goals of democratic legitimacy and may play a role in the process of reflective equilibrium [23]. Our aim was to examine general intuitions and compare them with theoretical models. We focused on resource allocation in newborn infants because of our clinical interest in newborn intensive care, but also because it allowed us to set aside differences in clinical need as well as the question of age in allocation [24]. We hypothesized that respondents would be inclined to give treatment preference to infants with a better predicted outcome, and that ethical inclinations may be associated with demographic characteristics, personality traits and political views.

## Methods

Members of the general public were recruited via an online survey platform (Mechanical Turk, Amazon, Seattle WA). The survey population platform has a stable and diverse pool of participants spanning a wide range of age, socio-economic and ethnic backgrounds [25]. Respondents were remunerated $1 for participation. Only those with a pre-existing survey approval rate of 95 % or above on greater than 100 tasks could participate.

Demographic questions assessed respondent age, gender, parental status, marital status, highest level of education, country of residence and religiosity.

The questionnaire consisted of 20 hypothetical rationing scenarios. Scenarios were piloted with a group of university students with non-medical backgrounds to test comprehensibility and clarity. SurveyMonkey (www.surveymonkey.com/) was used for survey design and data collection.

**Fig. 1:**
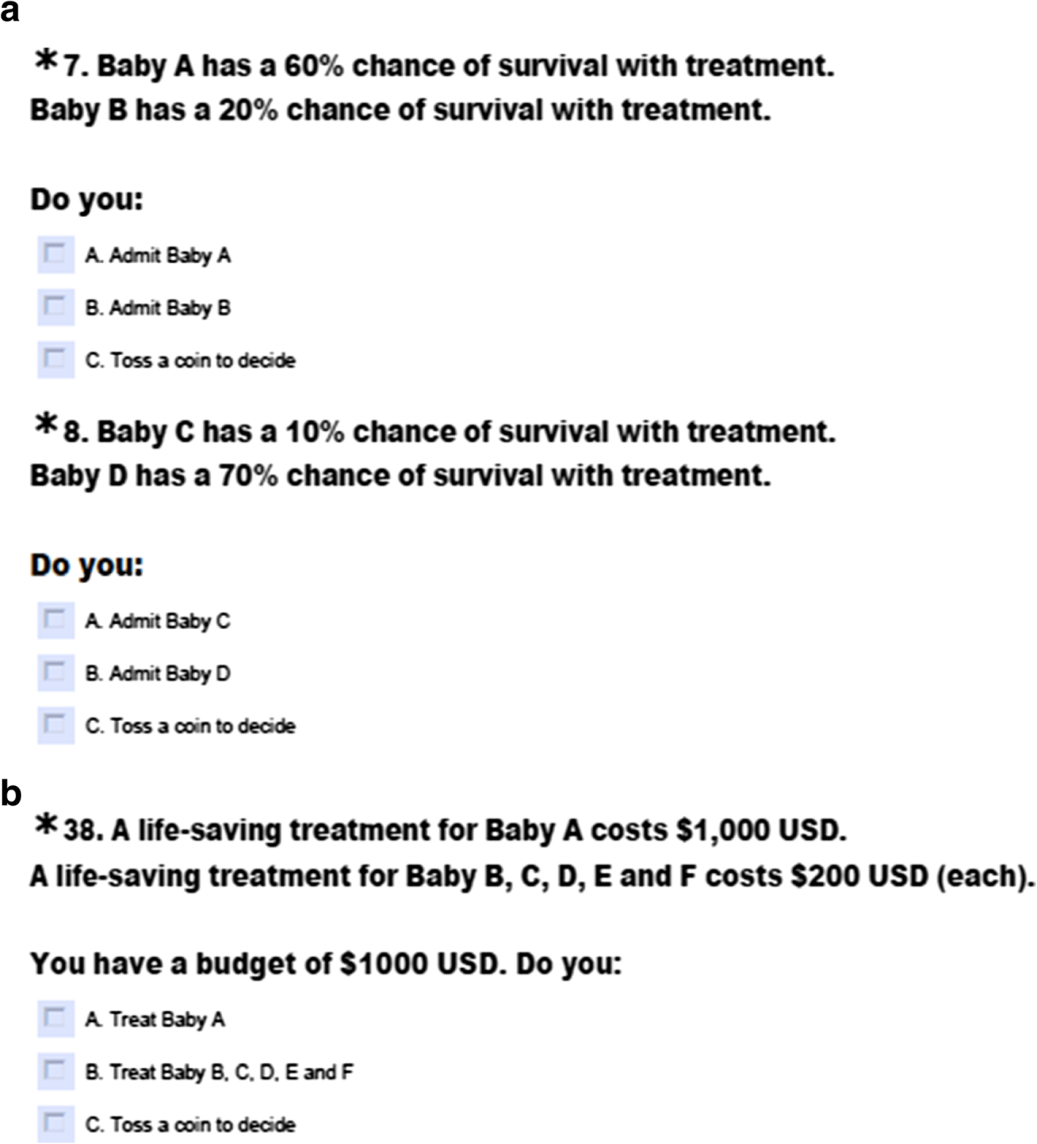
Hypothetical rationing scenarios in the setting of scarce neonatal intensive care resources. **a**. Example question with varying chance of survival. **b**. Example question with varying cost of treatment

Respondents were first asked to indicate their willingness to provide treatment for infants with different prognoses in a setting without any limit in bed capacity or resources (Likert scale: strongly disagree - strongly agree, 1–6). They were then required to consider a situation of scarcity with only one intensive care bed available and choose one of two critically ill newborn infants to admit for life-saving treatment. Respondents were informed that an infant not admitted would likely die. Infants varied to different degrees in either chance of survival, severity of predicted disability, life expectancy, or cost of treatment (Additional file 1: Questionnaire), as shown in Fig. 1a. Where cost varied between infants, an explicit fixed budget was specified, and less expensive treatments were described as leading to more lives saved (Fig. 1b). Available options were to A) admit the infant with the better outcome, B) admit the infant with the worse outcome, or C) toss a coin to decide. For analysis, Option A was classified as a *utilitarian* response, while Option C was classified an *egalitarian* response. Option B was designated N/A, as it did not correlate with any particular principle of allocation. The order of options varied randomly between questions.

Two scenarios were repeated in a version designed to emulate a hypothetical ‘veil of ignorance’ (Additional file 1), with choices potentially affecting one of their own future children [26]. Respondents were required to choose between a policy that would respond to situations of scarcity in the NICU either by admitting children with better-predicted outcome, or by tossing a coin to decide. They were asked to imagine that they would in the future have a newborn infant in need of intensive care, without knowing whether their child has the better or worse prognosis.

In order to examine the influence of underlying personality traits and political views on resource allocation preferences, three validated scales were also included: the Need for Cognition scale (a measure of tendency to enjoy effortful cognitive endeavours) [27], Empathic Concern index (a quantitative measure of empathy) [28], and the Social and Economic Conservatism scale (a measure of political ideology) [29].

A final question gauged response to a frequently cited philosophical thought experiment involving a choice between sending a lifeboat to rescue a sinking vessel containing one passenger, or one containing five passengers [30].

For sample size, we calculated that a sample of 85 would have a power of 80 % to detect a 20 % shift from egalitarian to utilitarian views and minimal-to-no switch between other response categories (*p* = 0.05) [29]. We assumed a 20 % rate of incomplete responses, and hence aimed for a total sample of 100–110 participants.

Data organisation, recoding and analysis were performed using SPSS Statistics version 22.0 software. In order to examine associations between utilitarian or egalitarian responses and demographic and personality factors, we examined a subset of scenarios with the most divergent responses (“Key indicator questions”: 40 % vs. 51 % chance of survival, none vs. mild disability, 40 vs. 41 years life expectancy, and treating 1 vs. 2 newborn infants). Likert responses were classified into three groups (strongly/moderately disagree, mildly disagree or mildly agree, strongly/moderately agree). Responses to scenarios involving allocation of limited resources were analysed using a 2x3 McNemar-Bowker Test for symmetry of responses on pairs of questions [31, 32, 34]. The McNemar-Bowker test of symmetry is used with matched pair, nominal data (with more than two categories) to assess whether changes in responses are significantly different. Unlike the chi-square test, the McNemar-Bowker test does not assume that observations are independent. It therefore accounts for the repeated measures nature of the survey design.

We calculated Cramer’s V, which provided a measure of the strength of association between paired responses. The lower the value of V, the more responses vary between a pair of questions. We used a cut-off value of V < 0.5 indicating that responses are at most moderately associated, and that there was a statistically larger difference in response to paired questions.

Independent samples t tests were conducted to compare the mean personality scores of utilitarian and egalitarian respondents and their responses to key indicator questions. Finally, Fisher’s exact test was used to find any association between demographic characteristics and allocation preferences. The null hypothesis was rejected at *p* < 0.05.

The University of Oxford Central University Research Ethics Committee and the Monash University Human Research Ethics Committee approved the study. Participation was voluntary, anonymous and limited to those 18 years or over.

## Results

One hundred and nine respondents completed the questionnaire. The demographic characteristics of respondents are summarized in Table 1. The mean age of respondents was 40 years. Respondents were predominantly female (61.5 %), 50 % had a tertiary education, and 56 % were religious. The majority (87 %) was from the US.

**Table 1:** Demographic characteristics of respondents, *N* = 109

Age	Mean ± SD, range	40.1 ± 11.6, 23.0–69.0
Sex	Male	42 (38.5 %)
	Female	67 (61.5 %)
Parental status	Parent	64 (58.7 %)
	Non-parent	45 (41.3 %)
Marital status	Single	55 (50.5 %)
	Married/De Facto	54 (49.5 %)
Highest level of education	Primary	3 (2.8 %)
	Secondary	52 (47.7 %)
	Tertiary	54 (49.5 %)
Religiosity	Non-religious (scores 1–3)	49 (41.1 %)
Scale of 1–7: 1 = strongly disagree, 7 = strongly agree	Neutral (score of 4)	8 (6.7 %)
	Religious (scores 5–7)	52 (43.8 %)
Country of origin	United States (US)	95 (87.2 %)
	Non-US	14 (12.8 %)

### Willingness to admit patients in the absence of scarcity

In the absence of scarcity, the majority of respondents indicated a willingness to provide treatment for infants in all scenarios, except in the setting of severe disability (42.7 % strongly or moderately agreed to admit the infant to intensive care) or highly expensive treatment ($7,000,000 cost, 49.6 % strongly or moderately agreed to admit), as shown in Table 2. Respondents expressed a stronger inclination to admit patients with a better prognosis. The higher the chance of survival, the more willing respondents were to admit the patient (*F*(1.2, 129.9) = 57.37, *p* <0.000). Similarly, respondents were more willing to admit patients with less severe future disability (*F*(1.5, 159.5) = 67.67, *p* < 0.000), longer life expectancy (*F*(1.3, 138.1) = 41.65, *p* < 0.000) or less expensive treatment (*F*(1.9, 200.3) = 63.16, *p* < 0.000).

**Table 2:** Willingness to admit patients based on prognostic variables in the absence of limited resources, *N* = 109

Prognostic variable	Disagreement (scores 1–2)	No strong opinion (scores 3–4)	Agreement (scores 5–6)
2A. Chance of survival			
10 %	8.1 %	26.8 %	65.2 %
20 %	5.1 %	30.3 %	64.3 %
60 %	0.0 %	5.4 %	94.6 %
70 %	0.0 %	1.8 %	98.3 %
2B. Severity of future disability			
Mild	1.8 %	12.7 %	85.4 %
Moderate	4.5 %	32.7 %	62.7 %
Severe	20.0 %	37.3 %	42.7 %
2C. Life expectancy			
5 years	12.8 %	27.3 %	60.0 %
15 years	3.6 %	24.5 %	71.9 %
25 years	1.8 %	13.6 %	84.6 %
2D. Cost of treatment			
$5,000 USD	0.0 %	4.6 %	95.4 %
$10,000 USD	0.0 %	7.3 %	92.7 %
$150,000 USD	3.7 %	15.6 %	80.7 %
$200,000 USD	7.4 %	15.6 %	77.1 %
$7,000,000 USD	18.4 %	32.1 %	49.6 %

### Limited resources and allocation

**Fig. 2:**
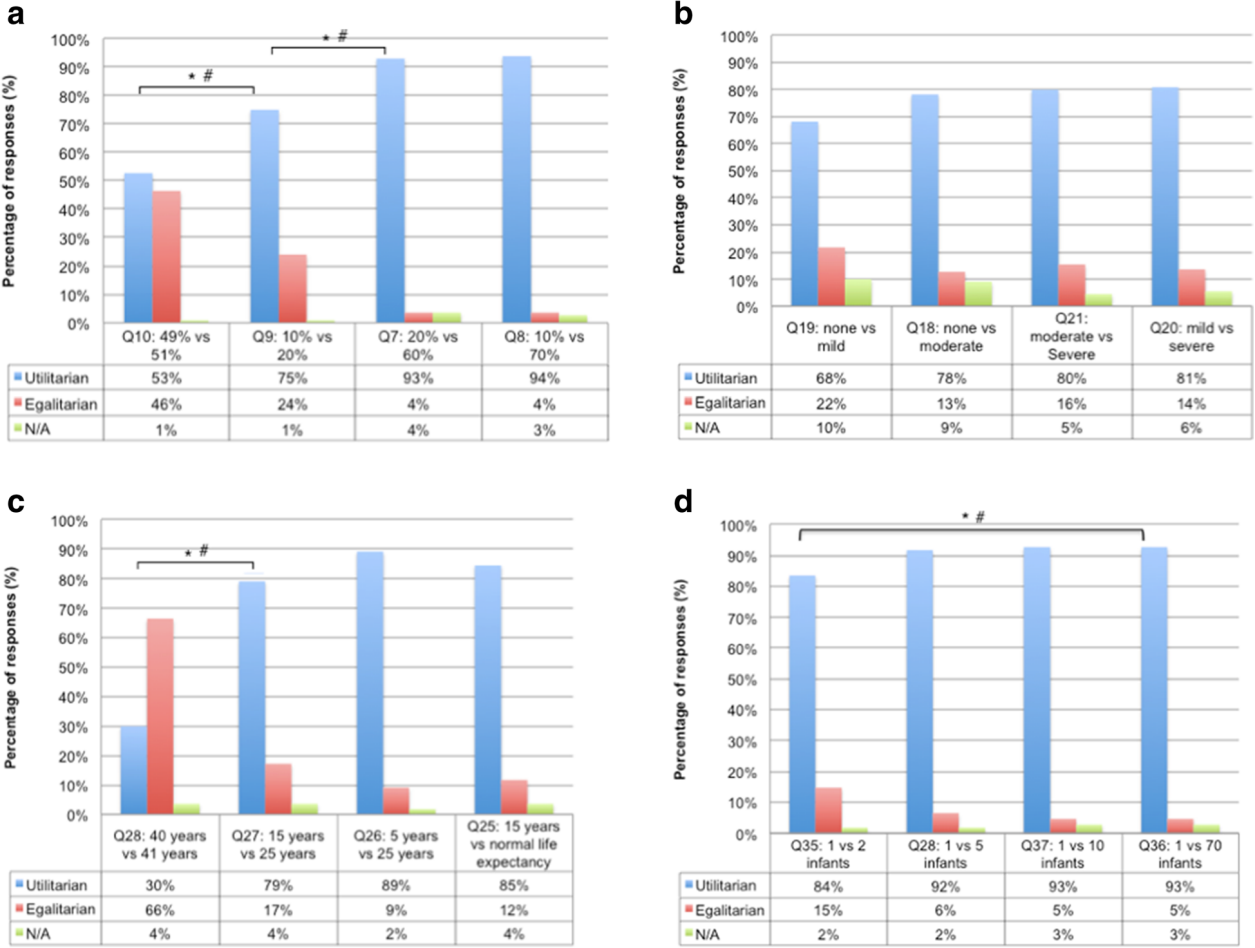
Responses to trade-off questions comparing infants with different prognostic variables: **a** chance of survival, **b** severity of predicted disability, **c** life expectancy, **d** cost of treatment

When forced to choose between patients because of limited resources, the majority of respondents chose to direct treatment to the infant with the better predicted outcome or lower cost of treatment (Fig. 2). The only exception was a scenario in which one infant was predicted to survive for 40 years, while the second was predicted to survive for 41 years. Two thirds of respondents elected to toss a coin to decide between the two infants in that scenario.

There was a significantly higher rate of utilitarian responses in scenarios with greater difference in predicted chance of survival, predicted duration of survival, or cost of treatment (Figure 2a, c and d). For example, in relation to predicted chance of survival, there was a significant asymmetry in responses to questions 10 and 9 (Q10 49 vs. 51 %, Q9 10 vs. 20 %, *X* ^2^ = 24.154, *P* < 0.000, Cramer’s V = 0.406) and questions 9 and 7 (Q9 10 % vs. 20 %, Q7 20 % vs. 60 %, *X* ^2^ = 25.000, *P* < 0.000, Cramer’s V = 0.424). There was also asymmetric distribution of responses for questions related to duration of survival (Fig. 2c) and cost of treatment (Fig. 2d). There were, however, no significant differences in the proportion of utilitarian responses between different degrees of disability (Fig. 2b).

There was no statistically significant difference between responses to questions in standard format and veil of ignorance versions (Table 3).

**Table 3:** Responses to standard format and veil of ignorance questions relating to two prognostic variables

Severity of disability: moderate vs. severe			
Standard format		Veil of ignorance format	
Utilitarian	80 %	Utilitarian	77 %
Egalitarian	16 %	Egalitarian	18 %
N/A	5 %	N/A	5 %
Chance of survival: 20 vs. 60 % chance			
Standard format		Veil of ignorance format	
Utilitarian	93 %	Utilitarian	89 %
Egalitarian	4 %	Egalitarian	7 %
N/A	4 %	N/A	4 %

In the lifeboat question, 98 % of respondents (88/90) elected to save five drowning people rather than one person: only 2 % elected to toss a coin to decide.

Personality traits did not appear to influence responses. There was no significant difference between the Empathic Concern scores of those who gave utilitarian responses and those who gave egalitarian responses to key indicator questions. There was also no significant difference between Need for Cognition scores or Social Economic Conservatism scale scores for utilitarian and egalitarian respondents.

Utilitarian responses to scenarios involving scarcity (i.e. where only one intensive care bed was available) were correlated with responses to questions in the absence of scarcity. The greater a respondent’s utilitarian propensity, the greater the effect of cost of treatment (*F*(29.5, 183.2) = 1.56, *p* < 0.05), chance of survival (*F*(18.1, 112.4) = 1.88, *p* < 0.05) and life expectancy (*F*(20.2, 125.0) = 1.73, *p* < 0.05) on inclination to admit the patient (in the non-scarce scenarios). Conversely, the willingness of respondents who were less utilitarian to admit patients remained similar regardless of prognostic information.

Some demographic characteristics influenced responses. Female respondents were significantly more inclined than male respondents to choose the egalitarian option of tossing a coin to determine which patient to admit when the trade-off was between a newborn infant with a mild disability and a newborn infant with no disability (*p* < 0.01). Parents were significantly more likely than non-parents to give an egalitarian response when asked to choose between treating one newborn infant or two (*p* = 0.01). Religious respondents were significantly more inclined to choose the utilitarian option of admitting a newborn infant with a 51 % chance of survival, over an infant with a 49 % chance (*p* = 0.02). Age, marital status, highest level of education, and country of origin had no significant association with allocation preferences.

## Discussion

This study examined the responses of a sample of the general public to a series of rationing dilemmas in newborn intensive care. When asked to choose between critically ill infants, the majority of respondents, in all but one scenario, sought the greatest benefit of treatment (i.e. chose the utilitarian option). This cross section of the lay public was remarkably utilitarian. Respondents were more likely to give utilitarian responses where there was a larger difference in predicted outcome between critically ill patients. Personality traits and political preferences were not associated with responses to rationing dilemmas.

Respondents in our survey consistently gave priority for treatment to patients with higher chance of survival, greater life expectancy, lower severity of disability and lower cost of treatment. Only in a scenario with a small (one year) difference in life expectancy did the majority of respondents choose to toss a coin to decide which patient to admit.

### Implications for ethical debate

Empirical findings like these cannot be used deductively to yield normative conclusions [33]. However, they may contribute to a dynamic process of reflective equilibrium [23]. Our study results provide valuable data on the relationship between the intuitions of the general public and ethical arguments.

The strikingly utilitarian tendency of the general public in this study is consistent with previous studies on healthcare practitioner resource allocation preferences [16, 18, 19, 22], however, contrasts with previous studies of the general public, which have indicated a preference for a more egalitarian approach [35–38].

One difference between this and previous rationing surveys is the requirement for respondents to choose between two individual patients in need of life-saving treatment, rather than choosing between two groups of patients, or different types of treatment. For example, in a survey conducted in Norway and repeated in the US, participants were asked to distribute an increase in health funding between the treatment of two illnesses of different severity [36, 38]. Participants gave priority to patients with more severe illness, even if they would benefit less from treatment. Life-or-death rationing at the cot-side in intensive care may appear closer to an emergency triage situation than prioritizing funding or health policy decisions [39]. It may be that utilitarian intuitions are stronger where the outcome for individual patients is more explicit.

One significant factor influencing NICU resource allocation decisions in our survey was the degree of difference between predicted outcomes in competing patients. This finding was consistent with a previous survey by Ubel et al., which investigated whether the public prioritizes equity or efficiency in distributing scarce organs to children needing a liver transplant [40]. That study found that respondents were less likely to give patients an equal opportunity for receiving treatment where there was a larger difference in prognosis between transplant candidates [35]. In our study, the same trend was seen in relation to three of the variables investigated. In a choice between infants with a 49 and 51 % chance of survival, a bare majority of respondents (52.7 %) chose the utilitarian option of treating the newborn infant with a higher chance of survival. In contrast, where there was a 10 % difference in chance of survival, three quarters of respondents elected to admit the infant with better prognosis.

Our study did not investigate the reasons why participants gave different responses. The largest number of egalitarian responses were seen when the discrepancy between outcomes was small; perhaps the difference in lifespan was considered by respondents to have negligible normative value. Alternatively, it may be that respondents were skeptical about the ability of clinicians to accurately predict the length of survival in adult life. Uncertainty about predictions might support a more egalitarian approach [41]. Finally, our results could be consistent with an ethical approach that balances a number of different ethical principles including equality and utility [40, 42]. Equality might be thought to outweigh small gains in utility, and lead to a different response in marginal cases.

Interestingly, three of the prognostic factors (in the absence of scarcity) showed a significant interaction with respondents’ utilitarian propensity to scenarios where resources were limited. The exception was for information about severity of future disability, which suggests that such information may affect a respondents’ inclination to admit regardless of utilitarian propensity.

### Implications for ethical theory

The results of this paper may also be of value for ethical theory. We gave our respondents a version of a much-discussed philosophical example, where they must choose between sending a lifeboat to save five people or one person [30]. Egalitarian philosophers have claimed that in such a situation we ought to toss a coin to decide (though acknowledge that this is counterintuitive) [30]. However, to our knowledge, the views of the general public about this have never been elicited. The overwhelming majority of respondents in our survey chose to send the lifeboat to save the larger number.

Political philosopher John Rawls famously described a procedure for developing fair and just public policy [26]. He imagined a group of hypothetical rational decision-makers who would have to decide how society should be structured without knowing their place in that society – whether they would be rich or poor, healthy or unhealthy, and so on. Rawls’ ‘veil of ignorance’ is designed to overcome prejudice or bias and is often thought to favour those who are worst off. Although Rawls did not apply this decision-making procedure to health care resource allocation, philosophers have taken different views about the sort of allocation policy that would be chosen behind the veil. For example, Singer et al. propose that in a scenario where two people need a life-saving treatment, but only one can receive it, a rational egoist would assume they have equal chance of being either person, and that they would maximize their own chances by directing treatment to the patient with better prognosis [43]. In contrast, Harris suggests that decision-makers behind the veil are likely to be risk-averse and would focus on reducing the chance for the individual at the time of allocation of the worst outcome (death) [44]. Harris argues that this would be accomplished by an egalitarian approach of random allocation (for example, tossing a coin) [44].

There have been few empirical studies of the impact of the veil of ignorance on public views about resource allocation [45]. We asked our respondents to imagine making policy about resource allocation in intensive care that would affect their own children (but without knowing their child’s prognosis). Respondents were predominantly utilitarian in their responses behind the veil. This appears to support Singer et al’s predictions [43]. 89.3 and 77.3 % of respondents chose the infant with a greater chance of survival and infant with a less severe disability, respectively. There were no statistically significant differences between responses to the policy questions and their equivalent versions without the veil. This finding does not resolve the debate between utilitarianism and egalitarianism, but does imply that the veil of ignorance thought experiment would favour the former (at least in situations where patients would be equally badly off without treatment).

### Demographic characteristics

There were some associations between demographic characteristics and responses to the survey. Parents were more egalitarian than non-parents in allocating resources based on cost of treatment. This is consistent with a survey of mothers of NICU infants from South Africa, in which the majority rejected rationing of resources entirely [22]. Females were more inclined to be egalitarian than males in relation to allocation of resources based on severity of disability. Religiosity influenced resource allocation preference based on chance of survival. Interestingly, respondents who described themselves as religious were more likely to give a utilitarian response than non-religious people. This finding is in contrast with previous studies, where a high proportion of people who chose egalitarian allocation were religious [46].

There was no relationship between age, marital status, or highest level of education and resource allocation preference. In a previous Australian study of the general public’s resource allocation preferences, all of these three factors were seen to influence treatment decisions [47].

### Personality tests

The mean scores of respondents on the Empathic Concern portion of the Interpersonal Reactivity Index, the Need for Cognition scale and the 12-item Social and Economic Conservatism Scale (SECS), had no relationship with their resource allocation inclinations. This might reflect that resource allocation preferences are not influenced by level of empathy or a greater level of deep thinking, but rather depend upon context-dependent moral evaluations.

### Strengths and limitations

This study is distinctive in assessing the views of a sample of the general public, rather than healthcare practitioners on resource allocation in the NICU. It provides valuable comparative data on responses to philosophical examples that have been widely discussed, but not previously studied empirically. The results of the survey should be taken cautiously, however. Our small online sample may not be representative of the wider general public. Mechanical Turk samples have been shown to be more diverse than convenience samples of students or the adult population, but less representative than face-to-face population sampling [47].

We were not able to determine why respondents chose particular answers, and responses may reflect uncritical initial responses that might change with further time to consider, or might be sensitive to the way in which cases were presented. Our scenarios specified equal starting points and equal clinical need for infants needing intensive care, therefore did not test prioritarian intuitions. Furthermore, in order to isolate factors that influenced decisions, scenarios were necessarily somewhat unrealistic and compared single variables sequentially. It is possible that a larger sample and factorial survey design would have allowed analysis of the interaction between variables in more complex real-world allocation scenarios [48].

## Conclusion

In this study, when faced with hypothetical difficult choices between critically ill neonates, respondents were strikingly utilitarian in their resource allocation preferences.

We focused on newborn infants; however, the principles that apply to rationing in newborn intensive care would also potentially apply in paediatric or adult intensive care. Future research should explore responses to rationing dilemmas in older children, adults and other populations. It would be helpful to compare prioritarian with utilitarian intuitions in rationing dilemmas. It would also be helpful to further clarify the decision-making rationale of the general public by using a qualitative methodology that would assess the reasons behind judgements, to use a factorial survey design that would permit analysis of the influence and interaction of different factors, and to assess whether the way in which cases are presented influences responses.

Although the findings of this study should not in themselves determine the practice of healthcare practitioners or the development of micro-allocation policy in the NICU, they can contribute to fruitful discussion in this area. In practice, clinicians should provide the best treatment that they can to newborn infants within available resources. Where possible, this will mean avoiding the sort of stark choices described in this paper through redistributing available resources, arranging transport of patients to other centres, or advocating for greater resources. However, difficult decisions will sometimes remain, especially in resource-limited settings. The apparently utilitarian inclination of respondents may indicate that the general public would support a policy for resource allocation in the NICU that directs clinicians to preferentially treat patients with better prognosis. Where there are small differences in predicted outcome between patients competing for life-saving treatment, it may be important to give them equal chances for receiving treatment [41].

## Additional files

Additional file 1: Questionnaire. (PDF 533 kb)
Additional file 2: ANOVA Interaction. (PDF 69 kb)
Additional file 3: Oxford Ethics Approval. (PDF 215 kb)
Additional file 4: Monash Ethics Approval. (PDF 491 kb)
Additional file 5: Survey Monkey Dataset. (PDF 226 kb)

## Abbreviations

CEA: Cost-effectiveness analysis
DALY: Disability-adjusted life year
N/A: Not applicable
NICU: Neonatal intensive care unit
QALY: Quality-adjusted life year
SECS: Social and economic conservatism scale

## References

[1] Howson CP, Kinney MV, Lawn JE (2012). Born too soon: the global health action report on preterm birth. Chapter 5. World Health Organization.

[2] Singh J, Lantos J, Meadow W (2004). End-of-life after birth: death and dying in a neonatal intensive care unit. Paediatrics.

[3] Marlow N (2004). Neurocognitive outcome after very preterm birth. Arch Dis Child Fetal Neonatal Ed.

[4] Sayeed SA (2006). The Marginally Viable Newborn: Legal Challenges, Conceptual Inadequacies, and Reasonableness. J Law Med Ethics.

[5] Bawden K, Broadbent R, Cartwright D (2013). Report of the Australian and New Zealand Neonatal Network 2011.

[6] Parmanum J, Field D, Rennie J, Steer P (2000). National census of availability of neonatal intensive care. British Association for Perinatal Medicine. BMJ.

[7] Watson S, Arulampalam W, Petrou S (2015). The effects of a one-to-one nurse-to-patient ratio on the mortality rate in neonatal intensive care: a retrospective, longitudinal, population-based study. Arch Dis Child Fetal Neonatal Ed.

[8] Chien LY, Whyte R, Aziz K, Thiessen P, Matthew D, Lee SK, Canadian Neonatal Network (2001). Improved outcome of preterm infants when delivered in tertiary care centers. Obstet Gynecol.

[9] Marlow N, Bennett C, Draper ED, Hennessy EM, Morgan AS, Costeloe KL (2014). Perinatal outcomes for extremely preterm babies in relation to place of birth in England: the EPICure 2 study. Arch Dis Child Fetal Neonatal Ed.

[10] Moazam F, Lakhani M (1990). Ethical dilemmas of health care in the developing nations [abstract]. J Pediatri Surg.

[11] World Health Organisation. 2013. Preterm Birth – Fact Sheet N 363. World Health Organization*.* Last updated November 2014. Date accessed July 2015. Available from: http://www.who.int/mediacentre/factsheets/fs363/en/.

[12] Singer PA, Mapa J (1998). Dimensions for Health Executives: Ethics of Resource Allocation. Hosp Q.

[13] Emanuel EL, Wetheimer A (2006). Who should get influenza vaccine when not all can?. Science.

[14] Whitehead M (1994). Who cares about equity in the NHS?. BMJ.

[15] Persad G, Wetheimer A, Emanuel EJ (2009). Principles for allocation of scarce medical interventions. Lancet.

[16] Parfit D (1997). Equality and priority. Ratio..

[17] Rawls J (1985). Justice as Fairness: Political not Metaphysical. Philosophy and Public Affairs. Vol. 14.

[18] Janvier A, Leblanc I, Barrington KJ (2008). Nobody likes premies: The relative value of patients’ lives. J Perinatol.

[19] Janvier A, Leblanc I, Barrington KJ (2008). The best-interest standard is not applied for neonatal resuscitation decisions. Paediatrics.

[20] Peerzada JM, Richardson DK, Burns JP (2004). Delivery room decision-making at the threshold of viability. J Paediatrics.

[21] Ballard DW, Li Y, Evans J, Ballard RA, Ubel PA (2002). Fear of litigation may increase resuscitation of infants born near the limits of viability. J Paediatrics.

[22] Wainer S, Khuzwayo H (1993). Attitudes of mothers, doctors and nurses toward neonatal intensive care in a developing society. Paediatrics.

[23] Dunn M, Sheehan M, Hope T, Parker M (2012). Toward Methodological Innovation in Empirical Ethics Research. Camb Q Health Ethics.

[24] Lockwood M (1988). Quality of life and resource allocation. Roy Inst Philos Lect Series.

[25] Mason W, Suri S (2012). Conducting behavioral research on Amazon’s Mechanical Turk. Behav Res.

[26] Rawls J (1971). A theory of justice.

[27] Cacioppo JT, Petty RE (1982). The need for cognition. J Pers Soc Psychol.

[28] Davis MH (1983). A multidimensional approach to individual differences in empathy. JSAS Cat Sel Doc Psychol.

[29] Everett JAC (2013). The 12 Item Social and Economic Conservatism Scale (SECS). PLoS One.

[30] Taurek JM (1977). Should the numbers count?. Philos Publ Aff.

[31] McNemar Q (1947). Note on the sampling error of the difference between correlated proportions or percentages. Psychometrika.

[32] Bowker AH (1948). Test for symmetry in contingency tables. J Am Stat Assoc.

[33] Borry P, Shotsmans P, Dierickx K (2004). What is the role of empirical research in bioethical reflection and decision-making? An ethical analysis. Med Health Care Philos.

[34] Ltd STDTP (2014). Tables of Sample Size Requirement for McNemar’s Test. StatsToDo.

[35] Ubel OA, Lowenstein G (1996). Distributing scarce livers. Soc Sci Med.

[36] Nord E (1993). The trade-off between severity of illness and treatment effect in cost-value analysis of healthcare. Health Policy.

[37] Ubel PA, Scalon D, Loewenstein G (1996). Individual utilities are inconsistent with rationing choices: a partial explanation of why Oregon’s cost-effectiveness list failed. Med Decis Making.

[38] Ubel PA (1999). How stable are people’s preferences for giving priority to severely ill patients?. Soc Sci Med.

[39] Ubel PA, Goold S (1998). Does bedside rationing violate patients’ best interests? An Exploration of “Moral Hazard”. Am J Med.

[40] Ubel PA, Loewenstein G (1996). Public perceptions of the importance of prognosis in allocation of transplantable livers to children. Med Decis Making.

[41] Wilkinson D, Savulescu J (2014). Disability, discrimination and death: is it justified to ration life saving treatment for newborn infants?. Monash Bioeth Rev.

[42] Lindholm L, Rosen M, Emmelin M (1998). How many lives is equity worth? A proposal for equity adjusted years of life saved. J Epidemiol Community Health.

[43] Singer P, McKie K, Kuhse H, Richardson J (1995). Double jeopardy and the use of QALYs in health care allocation. J Med Ethics.

[44] Harris J (1996). Would Aristotle have played Russian roulette?. J Med Ethics.

[45] Anderrson F, Lyttkens CH (1999). Preferences for equity in health behind a veil of ignorance. Health Econ.

[46] Anderson M, Richardson J, McKie J (2011). The relevance of personal characteristics in health care rationing: what the Australian public thinks and why. Amer J Eco Soc.

[47] Berinsky AJ, Huber GA, Gabriel SL (2012). Evaluating online labor markets for experimental research: Amazon.com’s mechanical turk. Pol Anal.

[48] Muller-Engelmann M, Krones T, Donner-Banzhoff N (2008). Decision making preferences in the medical encounter – a factorial survey design. BMC Health Serv Res.

