# Supplementary material for: The Intensive Care Lifeboat: a survey of lay attitudes to rationing dilemmas in neonatal intensive care
Source: BMC Med Ethics. 2016 Nov 8;17:69. doi: 10.1186/s12910-016-0152-y (PMC5100211; doi:10.1186/s12910-016-0152-y)
Supplement: Additional file 1: — Questionnaire. (PDF 533 kb) [file 12910_2016_152_MOESM1_ESM.pdf]

Ethical Dilemmas in Newborn Babies

General Information

We appreciate your interest in participating in this questionnaire. Quickly read through these terms before agreeing to participate by ticking the 'yes' box below.

We are investigating how people think about ethical dilemmas that arise when caring for newborn babies. You will be given some medical scenarios to read, and then answer questions on. It should take about 25 minutes. This is not a test of your medical knowledge. No background knowledge is required.

Mechanical Turk payment: 1.00 USD

Your answers will be completely anonymous and kept confidential. Please note that your participation is voluntary. You may withdraw at any point during the questionnaire for any reason, before submitting your answers. However, we are only able to reimburse participants who complete the full survey. The data will be stored in a password-protected file and may be used in academic publications.

This questionnaire is for an Honours project. The principal researcher is Chavy Arora (medical student), attached to the Oxford Uehiro Centre for Practical Ethics in the Department of Philosophy at the University of Oxford. This project is being completed under the supervision of Professor Julian Savulescu, Doctor Dominic Wilkinson and Professor Michael Selgelid. This project has been reviewed by, and received ethics clearance through the University of Oxford Central University Research Ethics Committee.

What if there is a problem?

If you have a concern about any aspect of this project, please speak to the researcher (Chavy Arora - chavy.arora@philosophy.ox.ac.uk) or their supervisor (Julian Savulescu - julian.savulescu@philosophy.ox.ac.uk), who will do their best to answer your query. The researcher should acknowledge your concern within 10 working days and give you an indication of how they intend to deal with it. If you remain unhappy or wish to make a formal complaint, please contact the chair of the Research Ethics Committee at the University of Oxford (Chair, Social Sciences & Humanities Inter-Divisional Research Ethics Committee; Email: ethics@socsci.ox.ac.uk; Address: Research Services, University of Oxford, Wellington Square, Oxford OX1 2JD). The chair will seek to resolve the matter in a reasonably expeditious manner.

*** 1. Please note that you may only participate in this survey if you are 18 years of age or older.**

○ I certify that I am 18 years of age or over.

*** 2. If you agree to participate and have read the terms above, please check the relevant box below to get started.**

☐ Yes, I agree to take part.

☐ No, I do not wish to take part

Ethical Dilemmas in Newborn Babies

Part 1.1

In the following scenarios, we would like you to imagine you are a doctor in the Neonatal Intensive Care Unit (NICU) of a hospital in your country. The NICU is where medical care is provided for very ill newborn infants. Sometimes doctors face difficult decisions about who to provide treatment to when the NICU is already full. You will be required to make decisions about whether or not to admit newborn infants to provide life-saving treatment. If they are NOT admitted, they are likely to die. In ALL of these situations, the parents have requested life-saving treatment for their child.

***3. A newborn infant is born prematurely. With treatment, they have a 60% chance of surviving. Life-saving treatment would be inexpensive and the infant would have a good quality of life in the future if they survived.**

If there were a bed available, would you admit this baby to the NICU?

Strongly disagree	Disagree	Somewhat disagree	Somewhat agree	Agree	Strongly agree
○	○	○	○	○	○

***4. A newborn infant is born prematurely. With treatment, they have a 10% chance of survival. Life-saving treatment would be inexpensive and the infant would have a good quality of life in the future if they survived.**

If there were a bed available, would you admit this baby to the NICU?

Strongly disagree	Disagree	Somewhat disagree	Somewhat agree	Agree	Strongly agree
○	○	○	○	○	○

***5. A newborn infant is born prematurely. With treatment, they have a 70% chance of survival. Life-saving treatment would be inexpensive and the infant would have a good quality of life in the future if they survived.**

If there were a bed available, would you admit this baby to the NICU?

Strongly disagree	Disagree	Somewhat disagree	Somewhat agree	Agree	Strongly agree
○	○	○	○	○	○

***6. A newborn infant is born prematurely. With treatment, they have a 20% chance of survival. Life-saving treatment would be inexpensive and the infant would have a good quality of life in the future if they survived.**

If there were a bed available, would you admit this baby to the NICU?

Strongly disagree	Disagree	Somewhat disagree	Somewhat agree	Agree	Strongly agree
○	○	○	○	○	○

Part 1.2

There are two newborn infants who have been born prematurely around the same time and both require NICU admission. There is only one bed available, so only one newborn can be admitted. The other newborn infant is likely to die. In all of these situations, the parents have requested life-saving treatment for their child.

***7. Baby A has a 60% chance of survival with treatment.**
Baby B has a 20% chance of survival with treatment.

Do you:

- [ ] A. Admit Baby A
- [ ] B. Admit Baby B
- [ ] C. Toss a coin to decide

***8. Baby C has a 10% chance of survival with treatment.**
Baby D has a 70% chance of survival with treatment.

Do you:

- [ ] A. Admit Baby C
- [ ] B. Admit Baby D
- [ ] C. Toss a coin to decide

***9. Baby E has a 20% chance of survival with treatment.**
Baby F has a 10% chance of survival with treatment.

Do you:

- [ ] A. Admit Baby E
- [ ] B. Admit Baby F
- [ ] C. Toss a coin to decide

***10. Baby G has a 49% chance of survival with treatment.**
Baby H has a 51% chance of survival with treatment.

Do you:

- [ ] A. Admit Baby G
- [ ] B. Admit Baby H
- [ ] C. Toss a coin to decide

***11. Imagine now that in one year's time you will have a newborn baby who needs to be admitted to intensive care. At the same time there will be another baby in need of treatment.**
One of the babies (baby J) has a 60% chance of survival, while the other baby (baby K) has a 20% chance of survival.
You don't know whether your baby will be Baby J or Baby K.
However, you are able to vote now for a policy that will tell doctors what to do in situations like this.

Do you:

- ☐ A. Vote for Policy A to treat Baby J (with a 60% chance of survival)
- ☐ B. Vote for Policy B to treat Baby K (with a 20% chance of survival)
- ☐ C. Vote for Policy C to toss a coin to decide which baby to treat

Policy question

***12. You have indicated that you would like to flip a coin. Before casting your vote, the policymakers have provided some extra information about the probability of survival with each policy.**

The probability of your child dying depends on which policy is used. The probability of a child surviving with each policy is:
Policy A (treat baby J with a higher chance of survival): overall 30% chance of infant survival
Policy B (treat baby K with a higher chance of survival): overall 10% chance of infant survival
Policy C (toss a coin to decide which child gets treated): overall 20% chance of infant survival

Do you:

- [ ] A. Vote for Policy A (30% chance of infant survival)
- [ ] B. Vote for Policy B (10% chance of infant survival)
- [ ] C. Toss a coin to decide (20% chance of infant survival)

Part 2.1

The following newborn infants are born with genetic problems affecting the brain and other organs. With treatment, they have a 100% chance of survival, however they will have some degree of disability. In all of these situations, the parents have requested life-saving treatment for their child. Please answer the following questions.

***13. The first newborn infant will have a MODERATE learning and physical disability if they are admitted to NICU and provided with life-saving treatment. This means that they will have a problem with seeing, hearing or talking even with glasses or a hearing aid. They will learn schoolwork slowly and need special help. They will also need to use a walking aid to walk, and special equipment to eat, bathe, dress or use the toilet. They will also sometimes get angry, worried or sad without any cause.**

If there were a bed available, would you admit this baby to the NICU?

Strongly disagree	Disagree	Somewhat disagree	Somewhat agree	Agree	Strongly agree
○	○	○	○	○	○

***14. The second newborn infant will have a MILD learning and physical disability if they are admitted to the NICU and provided with life-saving treatment. This means that they will be able to see, hear and speak normally with the help of glasses and a hearing aid. They will learn schoolwork more slowly than the rest of the class. They will be able to walk without special equipment or the help of a person, but will have a limp. They will be able to eat, bathe, dress and use the toilet normally and independently, and will be happy and not worried most of the time.**

If there were a bed available, would you admit this baby to the NICU?

Strongly disagree	Disagree	Somewhat disagree	Somewhat agree	Agree	Strongly agree
○	○	○	○	○	○

15. The third newborn infant will have a MILD TO MODERATE learning and physical disability if they are admitted to the NICU and provided with life-saving treatment. This means that he or she will be able to see, hear and speak almost normally with the help of glasses and a hearing aid. She or he will learn schoolwork far more slowly than the rest of the class, and may have behavioural problems in class. She or he will be able to walk with a walking frame. If you are reading this text, please leave the answer to this question blank. She or he will be able to eat, bathe, dress and use the toilet normally and independently, and will be happy and not worried most of the time.

If there were a bed available, would you admit this baby to the NICU?

Strongly disagree	Disagree	Somewhat disagree	Somewhat agree	Agree	Strongly agree
○	○	○	○	○	○

***16. The fourth newborn infant will have a SEVERE learning and physical disability if they are admitted to NICU and provided with life-saving treatment. This means that they will be blind, deaf or unable to talk. They will learn schoolwork very slowly and need special help. Also, they will need help from another person to eat, bathe, dress and use the toilet, and occasionally be fretful, depressed, anxious and irritable without any cause.**

If there were a bed available, would you admit this baby to the NICU?

Strongly disagree	Disagree	Somewhat disagree	Somewhat agree	Agree	Strongly agree
○	○	○	○	○	○

Part 2.2

There are two newborn infants who have been born with genetic problems around the same time and both require NICU admission. There is only one bed available, so only one newborn infant can be admitted. The other newborn infant is likely to die. In all of these situations, the parents have requested life-saving treatment for their child. Please answer the following questions.

***17. Imagine that in one year's time you will have a newborn baby who needs to be admitted to intensive care. At the same time, there will be another baby in need of treatment.**

One of the babies (Baby A) has a 100% chance of survival but will have a moderate learning and physical disability, while the other baby (Baby B) has a 100% chance of survival and will have a severe learning and physical disability.

You don't know whether your baby will be Baby A or Baby B. Only one baby can be treated.
However, you are able to vote now for a policy that will tell doctors what to do in situations like this

Do you:

- ☐ A. Vote for Policy A to treat Baby A (moderate learning and physical disability)
- ☐ B. Vote for Policy B to treat Baby B (severe learning and physical disability)
- ☐ C. Vote for Policy C to toss a coin to decide which baby to treat

***18. Baby C has a 100% chance of survival and will be moderately disabled (refer to definition above).**
Baby D has a 100% chance of survival and will have no disability.

Do you:

- ☐ A. Admit Baby C
- ☐ B. Admit Baby D
- ☐ C. Toss a coin to decide

***19. Baby E has a 100% chance of survival and will be mildly disabled.**
Baby F has a 100% chance of survival and will have no disability.

Do you:

- ☐ A. Admit Baby E
- ☐ B. Admit Baby F
- ☐ C. Toss a coin to decide.

***20. Baby G has a 100% chance of survival and will be severely disabled.**
Baby H has a 100% chance of survival and will be mildly disabled.

Do you:

- ☐ A. Admit Baby G
- ☐ B. Admit Baby H
- ☐ C. Toss a coin to decide

***21. Baby J has a 100% chance of survival and will be moderately disabled.**
Baby K has a 100% chance of survival and will be severely disabled.

Do you:

- ☐ A. Admit Baby J
- ☐ B. Admit Baby K
- ☐ C. Toss a coin to decide

Policy question 2

Part 3.1

A newborn infant is born with genetic problems affecting the brain and other organs. With treatment, they have a 100% chance of surviving, however will have ongoing health problems. These health problems will affect their life expectancy. In all of these situations, the parents have requested life-saving treatment for their child. Please answer the following questions.

***22. This newborn infant will survive the newborn period, but die at 15 years of age if they are admitted to the NICU.**

If a bed were available, would you admit this baby to the NICU?

Strongly disagree	Disagree	Somewhat disagree	Somewhat agree	Agree	Strongly agree
○	○	○	○	○	○

***23. This newborn infant will survive the newborn period, but die at 5 years of age if they are admitted to the NICU.**

If a bed were available, would you admit this baby to the NICU?

Strongly disagree	Disagree	Somewhat disagree	Somewhat agree	Agree	Strongly agree
○	○	○	○	○	○

***24. This newborn infant will likely survive the newborn period, but die at 25 years of age if they are admitted to the NICU.**

If there were a bed available, would you admit this baby to the NICU?

Strongly disagree	Disagree	Somewhat disagree	Somewhat agree	Agree	Strongly agree
○	○	○	○	○	○

Part 3.2

There are two newborn infants who have been born with genetic problems around the same time and both require NICU admission. There is only one bed available, so only one newborn infant can be admitted. The other newborn infant is likely to die. In all of these situations, the parents have requested life-saving treatment for their child. Please answer the following questions.

***25. Baby A will survive with treatment and live to 15 years of age.**
Baby B will survive with treatment and live to normal adult life expectancy.

Do you:

- ☐ A. Admit Baby A
- ☐ B. Admit Baby B
- ☐ C. Toss a coin to decide

***26. Baby C will survive with treatment and live to 5 years of age.**
Baby D will survive with treatment and live to 25 years of age.

Do you:

- ☐ A. Admit Baby C
- ☐ B. Admit Baby D
- ☐ C. Toss a coin to decide

***27. Baby E will survive with treatment and live to 15 years of age.**
Baby F will survive with treatment and live to 25 years of age.

Do you:

- [ ] A. Admit Baby E
- [ ] B. Admit Baby F
- [ ] C. Toss a coin to decide

***28. Baby G will survive with treatment and live to 41 years of age.**
Baby H will survive with treatment and live to 40 years of age.

Do you:

- [ ] A. Admit Baby G
- [ ] B. Admit Baby H
- [ ] C. Toss a coin to decide

***29. Baby J is expected to live to 5 years of age if not admitted to the NICU. If admitted, Baby J's life expectancy will increase to 10 years.**
Baby K is expected to live to 10 years of age if not admitted to the NICU. If admitted, Baby K's life expectancy will increase to 16 years.

Do you:

- [ ] A. Treat Baby J
- [ ] B. Treat Baby K
- [ ] C. Toss a coin to decide

Ethical Dilemmas in Newborn Babies

Part 4.1

A newborn infant is born with health problems affecting a number of organs. With treatment, they have a 100% chance of surviving to a normal life expectancy of 70 years. However, this treatment is expensive. Note that the average cost of NICU care for one day is $1,500 USD. In all of these situations, the parents have requested life-saving treatment for their child. Please answer the following questions.

***30. This newborn infant will survive the newborn period if treated, but this life-saving treatment will cost $200,000 USD.**

If there were a bed available, would you admit this baby to the NICU?

Strongly disagree	Disagree	Somewhat disagree	Somewhat agree	Agree	Strongly agree
○	○	○	○	○	○

***31. This newborn infant will survive the newborn period if treated, but this life-saving treatment will cost $7,000,000 (7 million) USD.**

If there were a bed available, would you admit this baby to the NICU?

Strongly disagree	Disagree	Somewhat disagree	Somewhat agree	Agree	Strongly agree
○	○	○	○	○	○

***32. This newborn infant will survive the newborn period if treated, but this life-saving treatment will cost $10,000 USD.**

If there were a bed available, would you admit this baby to the NICU?

Strongly disagree	Disagree	Somewhat disagree	Somewhat agree	Agree	Strongly agree
○	○	○	○	○	○

***33. This newborn infant will survive the newborn period if treated, but this life-saving treatment will cost $150,000 USD.**

If there were a bed available, would you admit this baby to the NICU?

Strongly disagree	Disagree	Somewhat disagree	Somewhat agree	Agree	Strongly agree
○	○	○	○	○	○

***34. This newborn infant will survive the newborn period if treated, but this life-saving treatment will cost $5,000 USD.**

If there were a bed available, would you admit this baby to the NICU?

Strongly disagree	Disagree	Somewhat disagree	Somewhat agree	Agree	Strongly agree
◯	◯	◯	◯	◯	◯

Part 4.2

There are a number of newborn infants born around the same time with health problems affecting a number of organs. With treatment, they have a 100% chance of surviving to normal life expectancy of 70 years. However, this treatment is expensive. You have a limited budget, so it is not possible to provide treatment to all the infants. If an infant is not admitted, they will not receive treatment, and is likely to lie. In all of these situations, the parents have requested life-saving treatment for their child. Please answer the following questions.

***35. A life-saving treatment for Baby A costs $10,000 USD.**
A life-saving treatment for Baby B and Baby C costs $5,000 USD (each).

You have a budget of $10,000 USD. Do you:

- [ ] A. Treat Baby A
- [ ] B. Treat Baby B and C
- [ ] C. Toss a coin to decide

***36. A life-saving treatment for Baby A costs $7,000,000 (7 million) USD.**
A life-saving treatment for 70 babies costs $100,000 USD (each).

You have a budget of $7,000,000 (7 million) USD. Do you:

- [ ] A. Treat Baby A
- [ ] B. Treat 70 babies
- [ ] C. Toss a coin to decide

***37. A life-saving treatment for Baby A costs $150,000 USD.**
A life-saving treatment for Baby B, C, D, E, F, G, H, I, J and K costs $15,000 USD (each).

You have a budget of $150,000 USD. Do you:

- [ ] A. Treat Baby A
- [ ] B. Treat Babies B, C, D, E, F, G, H, I, J and K
- [ ] C. Toss a coin to decide

***38. A life-saving treatment for Baby A costs $1,000 USD.**
A life-saving treatment for Baby B, C, D, E and F costs $200 USD (each).

You have a budget of $1000 USD. Do you:

- [ ] A. Treat Baby A
- [ ] B. Treat Baby B, C, D, E and F
- [ ] C. Toss a coin to decide

Part 5: Demographic Questions

***39. What year were you born?**

Select a year

***40. What is your gender?**

- [ ] Male
- [ ] Female
- [ ] Other

***41. Are you a parent?**

- [ ] Yes
- [ ] No

***42. What is your nationality?**

***43. What country do you live in?**

***44. Are you religious?**

Strongly Disagree	Disagree	Somewhat Disagree	Neutral	Somewhat Agree	Agree	Strongly Agree
○	○	○	○	○	○	○

***45. What is your religion?**

***46. What is your marital status?**

- [ ] Single
- [ ] Married
- [ ] De Facto relationship

***47. What is your highest level of education?**

- [ ] Primary school education
- [ ] Secondary education
- [ ] Tertiary education

Ethical Dilemmas in Newborn Babies

Part 6: Lifeboat Dilemma

48. Imagine you are manning the sole coastguard boat on duty. Two boats have been overturned some distance from each other. There are 5 people on one life raft 50 miles due north, and 1 person on another life raft 50 miles due south. A storm is brewing and it is highly likely you will only be able to reach one life raft before the storm overturns them and the people drown.

Do you:

- ☐ A. Save the 5 people stranded on the life raft 50 miles due north
- ☐ B. Save the 1 person stranded on the life raft 50 miles due south
- ☐ C. Toss a coin to decide between option A and option B

Part 7

The following statements ask about your thoughts and feelings in a variety of situations. For each question, indicate how well it describes you by choosing the appropriate number on a scale from 1-5.

An answer of 1 means "Does not describe me well", and an answer of 5 means "Describes me very well".

***49. Read each item carefully before responding. Answer as honestly and accurately as you can. An answer of 1 (furthest to the left) means "Does not describe me well at all", and a response of 5 (furthest to the right) means "Describes me very well"**

	1	2	3	4	5
1. I often have tender, concerned feelings for people less fortunate than me.	○	○	○	○	○
2. Sometimes I don't feel very sorry for other people when they are having problems.	○	○	○	○	○
3. When I see someone being taken advantage of, I feel kind of protective towards them.	○	○	○	○	○
4. Other people's misfortunes do not usually disturb me a great deal.	○	○	○	○	○
5. When I see someone being treated unfairly, I sometimes don't feel very much pity for them.	○	○	○	○	○
6. I am quite often touched by things that I see happen.	○	○	○	○	○
7. I would describe myself as a pretty soft-hearted person	○	○	○	○	○

***50. The following statements ask about your thoughts and feelings in a variety of situations. For each item, show how well it describes you by choosing the appropriate number on the scale from -4 to +4.**

+ 4 = Very strong agreement

+3 = Strong agreement

+2 = Moderate agreement

+1 = Slight agreement

0 = Neither agreement nor disagreement

-1 = Slight disagreement

-2 = Moderate disagreement

-3 = Strong disagreement

-4 = Very strong disagreement

	-4	-3	-2	-1	0	+1	+2	+3	+4
1. I would prefer simple to complex problems.	◯	◯	◯	◯	◯	◯	◯	◯	◯
2. I like to have the responsibility of handling a situation that requires a lot of thinking.	◯	◯	◯	◯	◯	◯	◯	◯	◯
3. Thinking is not my idea of fun.	◯	◯	◯	◯	◯	◯	◯	◯	◯
4. I would rather do something that requires little thought than something that is sure to challenge my thinking abilities.	◯	◯	◯	◯	◯	◯	◯	◯	◯
5. I try to anticipate and avoid situations where there is likely a chance I will have to think in depth about something.	◯	◯	◯	◯	◯	◯	◯	◯	◯
6. I find satisfaction in deliberating hard and for long hours.	◯	◯	◯	◯	◯	◯	◯	◯	◯
7. I only think as hard as I have to.	◯	◯	◯	◯	◯	◯	◯	◯	◯
8. I prefer to think about small, daily projects to long-term ones.	◯	◯	◯	◯	◯	◯	◯	◯	◯
9. I like tasks that require little thought once	◯	◯	◯	◯	◯	◯	◯	◯	◯

I've learned them.

10. The idea of relying on thought to make my way to the top appeals to me.	○	○	○	○	○	○	○	○	○
11. I really enjoy a task that involves coming up with new solutions to problems.	○	○	○	○	○	○	○	○	○
12. Learning new ways to think doesn't excite me very much.	○	○	○	○	○	○	○	○	○
13. I prefer my life to be filled with puzzles that I must solve.	○	○	○	○	○	○	○	○	○
14. The notion of thinking abstractly is appealing to me.	○	○	○	○	○	○	○	○	○
15. I would prefer a task that is intellectual, difficult, and important to one that is somewhat important but does not require much thought.	○	○	○	○	○	○	○	○	○
16. I feel relief rather than satisfaction after completing a task that required a lot of mental effort.	○	○	○	○	○	○	○	○	○
17. It's enough for me that something gets the job done; I don't care how or why it works.	○	○	○	○	○	○	○	○	○
18. I usually end up deliberating about issues even when they do not affect me personally.	○	○	○	○	○	○	○	○	○

***51. Please indicate the extent to which you feel positive or negative towards each of the following 12 issues by ranking each issue with a number from 0 to 100.**

Scores of 0 indicate greater negativity, and scores of 100 indicate greater positivity. A score of 50 indicates that you feel neutral about the issue.

	Choose a number
1. Abortion	
2. Limited government	
3. Military and national security	
4. Religion	
5. Welfare benefits	
6. Gun ownership	
7. Traditional marriage	
8. Traditional values	
9. Fiscal responsibility	
10. Business	
11. The family unit	
12. Patriotism	
