# Supplementary material for: The Intensive Care Lifeboat: a survey of lay attitudes to rationing dilemmas in neonatal intensive care
Source: BMC Med Ethics. 2016 Nov 8;17:69. doi: 10.1186/s12910-016-0152-y (PMC5100211; doi:10.1186/s12910-016-0152-y)
Supplement: Additional file 2: — ANOVA Interaction. (PDF 69 kb) [file 12910_2016_152_MOESM2_ESM.pdf]

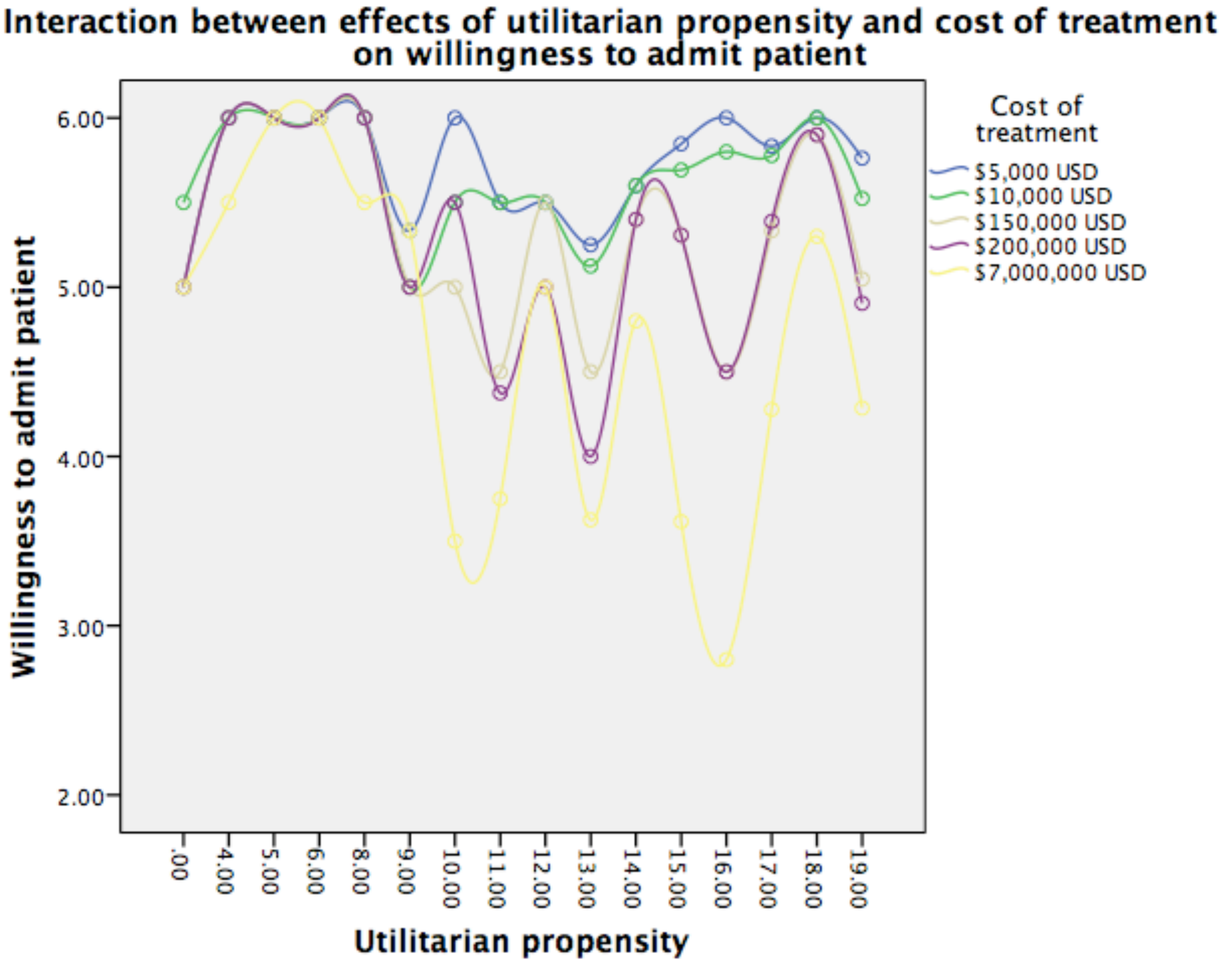
Interaction between effects of utilitarian propensity and cost of treatment on willingness to admit patient
Willingness to admit patient
6.00
5.00
4.00
3.00
2.00
.00
4.00
5.00
6.00
8.00
9.00
10.00
11.00
12.00
13.00
14.00
15.00
16.00
17.00
18.00
19.00
Utilitarian propensity
Cost of treatment
$5,000 USD
$10,000 USD
$150,000 USD
$200,000 USD
$7,000,000 USD
