# Supplementary material for: The Intensive Care Lifeboat: a survey of lay attitudes to rationing dilemmas in neonatal intensive care
Source: BMC Med Ethics. 2016 Nov 8;17:69. doi: 10.1186/s12910-016-0152-y (PMC5100211; doi:10.1186/s12910-016-0152-y)
Supplement: Additional file 3: — Oxford Ethics Approval. (PDF 215 kb) [file 12910_2016_152_MOESM3_ESM.pdf]

SOCIAL SCIENCES & HUMANITIES
INTER-DIVISIONAL RESEARCH ETHICS COMMITTEE

Research Services, University of Oxford, Wellington Square, Oxford OX1 2JD
Tel: +44(0)1865 616576 Fax: +44(0)1865 280467
ethics@socsci.ox.ac.uk

Co-ordinator of the SSH IDREC
Research Services

UNIVERSITY OF OXFORD

10 June 2014

Chavy Arora
Department of Philosophy

Dear Chavy,

Research Ethics Approval

Ref No: SSD/CUREC1A/14-110

Title: The Ethics of Resource Allocation in the Neonatal Intensive Care Unit: An Empirical Perspective

The above application has been considered on behalf of the Social Sciences and Humanities Inter-divisional Research Ethics Committee (IDREC) in accordance with the procedures laid down by the University for ethical approval of all research involving human participants.

I am pleased to inform you that, on the basis of the information provided to the IDREC, the proposed research has been judged as meeting appropriate ethical standards, and accordingly approval has been granted.

Should there be any subsequent changes to the project, which raise ethical issues not covered in the original application, you should submit details to the IDREC for consideration.

Yours sincerely,

KMHicks

Kate Hicks
Coordinator and Secretary SSH IDREC

cc: Julian Savulescu, Dominic Wilkinson, Michael Selgelid

KH/CK
