# Supplementary material for: The Intensive Care Lifeboat: a survey of lay attitudes to rationing dilemmas in neonatal intensive care
Source: BMC Med Ethics. 2016 Nov 8;17:69. doi: 10.1186/s12910-016-0152-y (PMC5100211; doi:10.1186/s12910-016-0152-y)
Supplement: Additional file 4: — Monash Ethics Approval. (PDF 491 kb) [file 12910_2016_152_MOESM4_ESM.pdf]

MONASH University

Monash University Human Research Ethics Committee (MUHREC)
Research Office

Human Ethics Certificate of Approval

This is to certify that the project below was considered by the Chair of the Monash University Human Research Ethics Committee. The Chair was satisfied that the proposal meets the requirements of the *National Statement on Ethical Conduct in Human Research* and has granted approval.

Project Number: CF14/1798 - 2014000906

Project Title: Ethics of Resource Allocation in the Neonatal Intensive Care Unit

Chief Investigator: Julian Saculescu

Approved: **From:** 30 June 2014 **To:** 30 June 2019

Terms of approval - ***Failure to comply with the terms below is in breach of your approval and the Australian Code for the Responsible Conduct of Research.***

1. Approval is only valid whilst you hold a position at Monash University and approval at the primary HREC is current.
2. **Future correspondence:** Please quote the project number and project title above in any further correspondence.
3. **Final report:** A Final Report should be provided at the conclusion of the project. MUHREC should be notified if the project is discontinued before the expected date of completion.
4. **Retention and storage of data:** The Chief Investigator is responsible for the storage and retention of original data pertaining to a project for a minimum period of five years.

Professor Nip Thomson
Chair, MUHREC

cc: Miss Chavy Arora

Postal – Monash University, Vic 3800, Australia
Building 3E, Room 111, Clayton Campus, Wellington Road, Clayton
Telephone +61 3 9905 5490 Facsimile +61 3 9905 3831
Email muhrec@monash.edu http://www.monash.edu.au/researchoffice/human/
ABN 12 377 614 012 CRICOS Provider #00008C
