# Supplementary material for: The Intensive Care Lifeboat: a survey of lay attitudes to rationing dilemmas in neonatal intensive care
Source: BMC Med Ethics. 2016 Nov 8;17:69. doi: 10.1186/s12910-016-0152-y (PMC5100211; doi:10.1186/s12910-016-0152-y)
Supplement: Additional file 5: — Survey Monkey Dataset. (PDF 226 kb) [file 12910_2016_152_MOESM5_ESM.pdf]

## Q1 Please note that you may only participate in this survey if you are 18 years of age or older.

Answered: 122 Skipped: 0

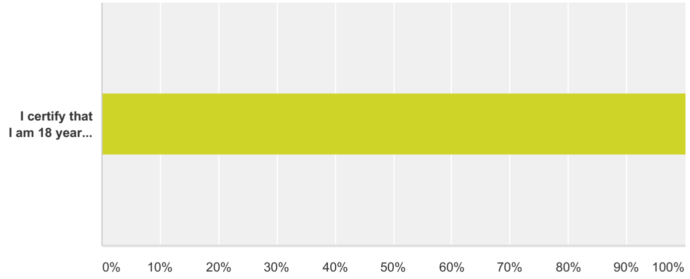


| Answer Choices | Responses | |
|---|---|---|
| I certify that I am 18 years of age or over. | 100.00% | 122 |
| Total | | 122 |

## Q2 If you agree to participate and have read the terms above, please check the relevant box below to get started.

Answered: 122 Skipped: 0

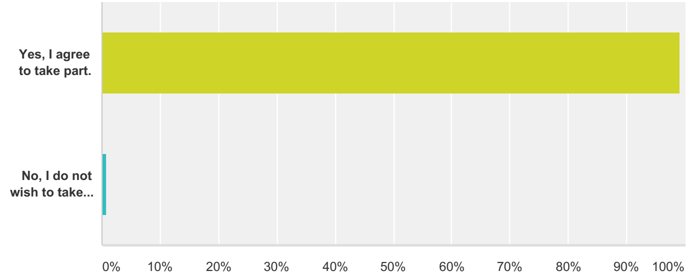


| Answer Choices | Responses | |
|---|---|---|
| Yes, I agree to take part. | 99.18% | 121 |
| No, I do not wish to take part | 0.82% | 1 |
| Total Respondents: 122 | | |

## Q3 A newborn infant is born prematurely. With treatment, they have a 60% chance of surviving. Life-saving treatment would be inexpensive and the infant would have a good quality of life in the future if they survived. If there were a bed available, would you admit this baby to the NICU?

Answered: 112 Skipped: 10

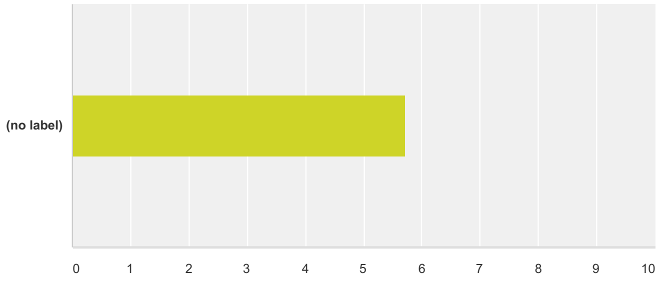


| | Strongly disagree | Disagree | Somewhat disagree | Somewhat agree | Agree | Strongly agree | Total | Weighted Average |
|---|---|---|---|---|---|---|---|---|
| (no label) | 0.00%<br>0 | 0.00%<br>0 | 0.00%<br>0 | 5.36%<br>6 | 18.75%<br>21 | 75.89%<br>85 | 112 | 5.71 |

## Q4 A newborn infant is born prematurely. With treatment, they have a 10% chance of

**survival. Life-saving treatment would be inexpensive and the infant would have a good quality of life in the future if they survived. If there were a bed available, would you admit this baby to the NICU?**

Answered: 112 Skipped: 10

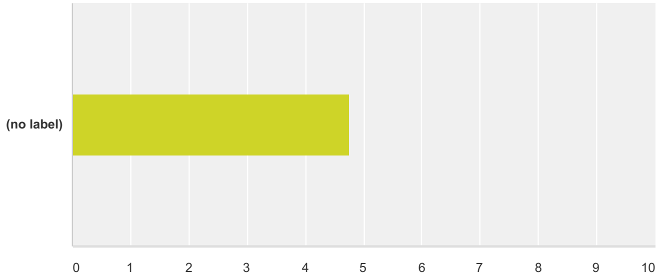


| | Strongly disagree | Disagree | Somewhat disagree | Somewhat agree | Agree | Strongly agree | Total | Weighted Average |
|---|---|---|---|---|---|---|---|---|
| (no label) | 3.57%<br>4 | 4.46%<br>5 | 11.61%<br>13 | 15.18%<br>17 | 22.32%<br>25 | 42.86%<br>48 | 112 | 4.77 |

## Q5 A newborn infant is born prematurely. With treatment, they have a 70% chance of survival. Life-saving treatment would be inexpensive and the infant would have a good quality of life in the future if they survived. If there were a bed available, would you admit this baby to the NICU?

Answered: 112 Skipped: 10

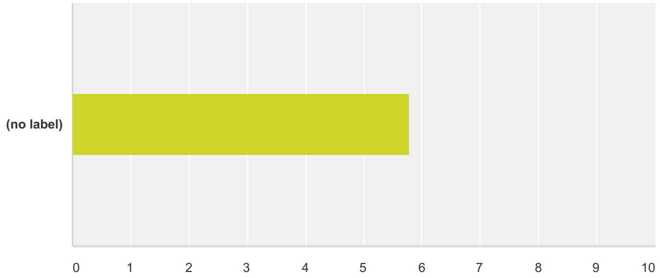


| | Strongly disagree | Disagree | Somewhat disagree | Somewhat agree | Agree | Strongly agree | Total | Weighted Average |
|---|---|---|---|---|---|---|---|---|
| (no label) | 0.00%<br>0 | 0.00%<br>0 | 0.00%<br>0 | 1.79%<br>2 | 17.86%<br>20 | 80.36%<br>90 | 112 | 5.79 |

## Q6 A newborn infant is born prematurely. With treatment, they have a 20% chance of survival. Life-saving treatment would be inexpensive and the infant would have a good quality of life in the future if they survived. If there were a bed available, would you admit this baby to the NICU?

Answered: 112 Skipped: 10

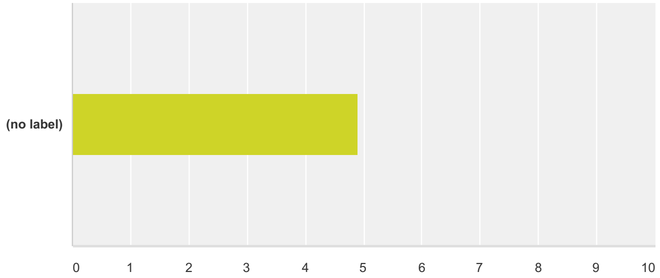


| | Strongly disagree | Disagree | Somewhat disagree | Somewhat agree | Agree | Strongly agree | Total | Weighted Average |
|---|---|---|---|---|---|---|---|---|
| (no label) | 0.89%<br>1 | 4.46%<br>5 | 8.04%<br>9 | 22.32%<br>25 | 18.75%<br>21 | 45.54%<br>51 | 112 | 4.90 |

## Q7 Baby A has a 60% chance of survival with treatment.Baby B has a 20% chance of survival with treatment.Do you:

Answered: 112 Skipped: 10

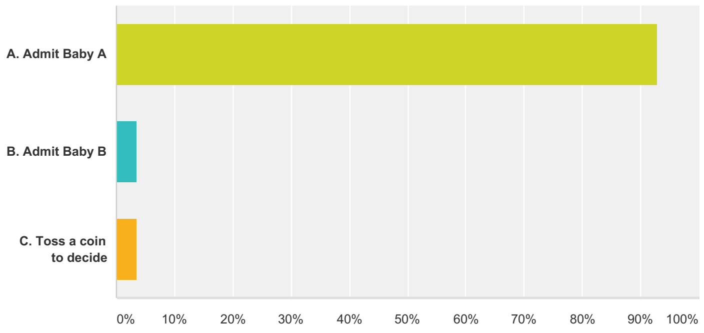


| Answer Choices | Responses | |
|---|---|---|
| A. Admit Baby A | 92.86% | 104 |
| B. Admit Baby B | 3.57% | 4 |
| C. Toss a coin to decide | 3.57% | 4 |
| Total Respondents: 112 | | |

## Q8 Baby C has a 10% chance of survival with treatment.Baby D has a 70% chance of survival with treatment.Do you:

Answered: 112 Skipped: 10

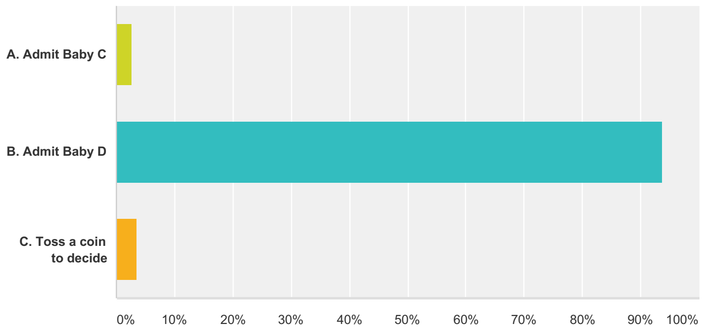


| Answer Choices | Responses | |
|---|---|---|
| A. Admit Baby C | 2.68% | 3 |
| B. Admit Baby D | 93.75% | 105 |
| C. Toss a coin to decide | 3.57% | 4 |
| Total Respondents: 112 | | |

## Q9 Baby E has a 20% chance of survival with treatment.Baby F has a 10% chance of survival with treatment. Do you:

Answered: 112 Skipped: 10

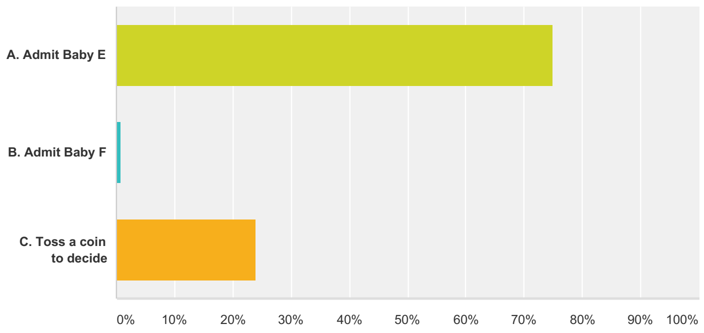


| Answer Choices | Responses |
|---|---|

| | | |
|---|---|---|
| A. Admit Baby E | 75.00% | 84 |
| B. Admit Baby F | 0.89% | 1 |
| C. Toss a coin to decide | 24.11% | 27 |
| Total Respondents: 112 | | |

## Q10 Baby G has a 49% chance of survival with treatment.Baby H has a 51% chance of survival with treatment.Do you:

Answered: 112 Skipped: 10

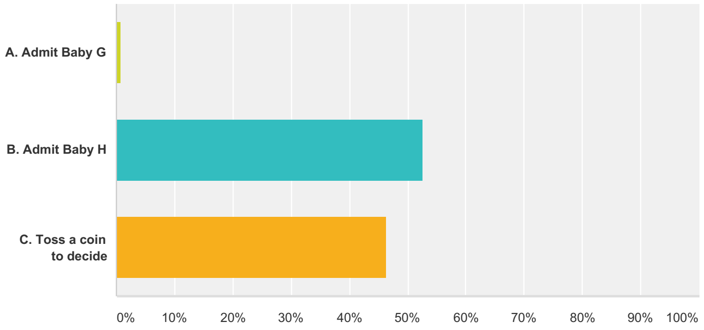


| Answer Choices | Responses | |
|---|---|---|
| A. Admit Baby G | 0.89% | 1 |
| B. Admit Baby H | 52.68% | 59 |
| C. Toss a coin to decide | 46.43% | 52 |
| Total Respondents: 112 | | |

## Q11 Imagine now that in one year's time you will have a newborn baby who needs to be admitted to intensive care. At the same time there will be another baby in need of treatment.One of the babies (baby J) has a 60% chance of survival, while the other baby (baby K) has a 20% chance of survival.You don't know whether your baby will be Baby J or Baby K.However, you are able to vote now for a policy that will tell doctors what to do in situations like this.Do you:

Answered: 112 Skipped: 10

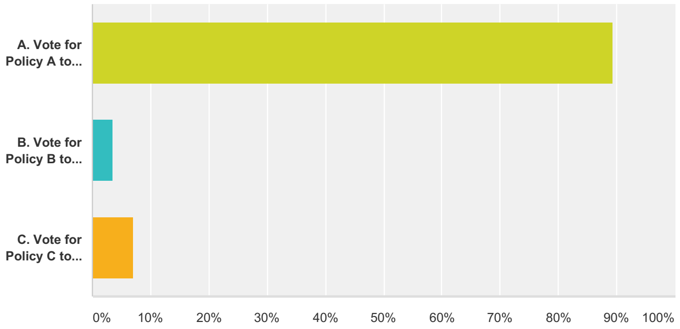


| Answer Choices | Responses | |
|---|---|---|
| A. Vote for Policy A to treat Baby J (with a 60% chance of survival) | 89.29% | 100 |
| B. Vote for Policy B to treat Baby K (with a 20% chance of survival) | 3.57% | 4 |
| C. Vote for Policy C to toss a coin to decide which baby to treat | 7.14% | 8 |
| Total Respondents: 112 | | |

## Q12 You have indicated that you would like to flip a coin. Before casting your vote, the policymakers have provided some extra information about the probability of survival with each policy.The probability of your

**child dying depends on which policy is used. The probability of a child surviving with each policy is:Policy A (treat baby J with a higher chance of survival): overall 30% chance of infant survivalPolicy B (treat baby K with a higher chance of survival): overall 10% chance of infant survivalPolicy C (toss a coin to decide which child gets treated): overall 20% chance of infant survivalDo you:**

Answered: 8 Skipped: 114

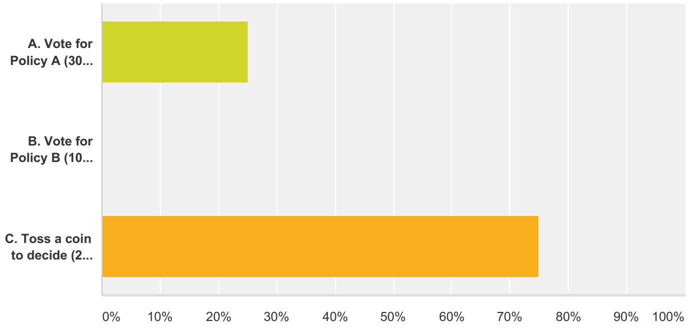


| Answer Choices | Responses | |
|---|---|---|
| A. Vote for Policy A (30% chance of infant survival) | 25.00% | 2 |
| B. Vote for Policy B (10% chance of infant survival) | 0.00% | 0 |
| C. Toss a coin to decide (20% chance of infant survival) | 75.00% | 6 |
| Total Respondents: 8 | | |

**Q13 The first newborn infant will have a MODERATE learning and physical disability if they are admitted to NICU and provided with life-saving treatment. This means that they will have a problem with seeing, hearing or talking even with glasses or a hearing aid. They will learn schoolwork slowly and need special help. They will also need to use a walking aid to walk, and special equipment to eat, bathe, dress or use the toilet. They will also sometimes get angry, worried or sad without any cause.If there were a bed available, would you admit this baby to the NICU?**

Answered: 110 Skipped: 12

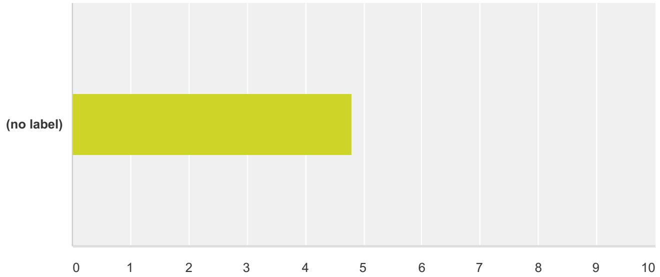


| | Strongly disagree | Disagree | Somewhat disagree | Somewhat agree | Agree | Strongly agree | Total | Weighted Average |
|---|---|---|---|---|---|---|---|---|
| (no label) | 3.64%<br>4 | 0.91%<br>1 | 10.00%<br>11 | 22.73%<br>25 | 22.73%<br>25 | 40.00%<br>44 | 110 | 4.80 |

**Q14 The second newborn infant will have a MILD learning and physical disability if they are admitted to the NICU and provided with life-saving treatment. This means that they will be able to see, hear and speak normally with the help of glasses and a hearing aid.**

**They will learn schoolwork more slowly than the rest of the class. They will be able to walk without special equipment or the help of a person, but will have a limp. They will be able to eat, bathe, dress and use the toilet normally and independently, and will be happy and not worried most of the time.If there were a bed available, would you admit this baby to the NICU?**

Answered: 110 Skipped: 12

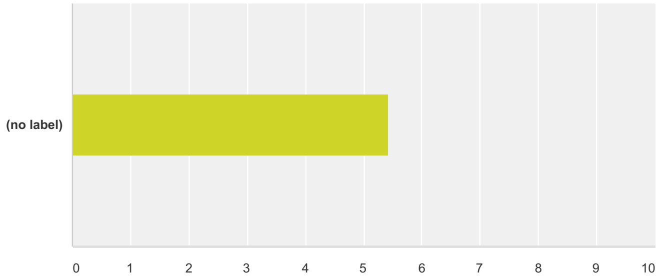


| | Strongly disagree | Disagree | Somewhat disagree | Somewhat agree | Agree | Strongly agree | Total | Weighted Average |
|---|---|---|---|---|---|---|---|---|
| (no label) | 0.91%<br>1 | 0.91%<br>1 | 0.91%<br>1 | 11.82%<br>13 | 22.73%<br>25 | 62.73%<br>69 | 110 | 5.43 |

**Q15 The third newborn infant will have a MILD TO MODERATE learning and physical disability if they are admitted to the NICU and provided with life-saving treatment. This means that he or she will be able to see, hear and speak almost normally with the help of glasses and a hearing aid. She or he will learn schoolwork far more slowly than the rest of the class, and may have behavioural problems in class. She or he will be able to walk with a walking frame. If you are reading this text, please leave the answer to this question blank. She or he will be able to eat, bathe, dress and use the toilet normally and independently, and will be happy and not worried most of the time. If there were a bed available, would you admit this baby to the NICU?**

Answered: 52 Skipped: 70

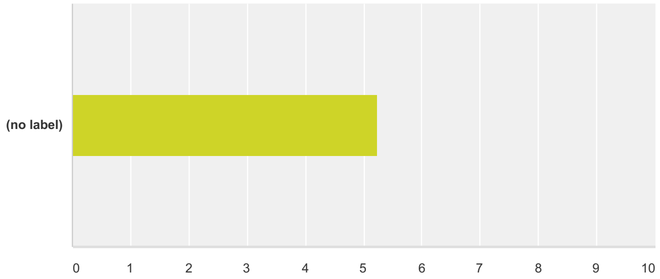


| | Strongly disagree | Disagree | Somewhat disagree | Somewhat agree | Agree | Strongly agree | Total | Weighted Average |
|---|---|---|---|---|---|---|---|---|
| (no label) | 0.00%<br>0 | 0.00%<br>0 | 3.85%<br>2 | 17.31%<br>9 | 28.85%<br>15 | 50.00%<br>26 | 52 | 5.25 |

**Q16 The fourth newborn infant will have a SEVERE learning and physical disability if they are admitted to NICU and provided with life-saving treatment. This means that they will be blind, deaf or unable to talk. They will learn schoolwork very slowly and need special help. Also, they will need help from another person to eat, bathe, dress and use**

**the toilet, and occasionally be fretful, depressed, anxious and irritable without any cause. If there were a bed available, would you admit this baby to the NICU?**

Answered: 110 Skipped: 12

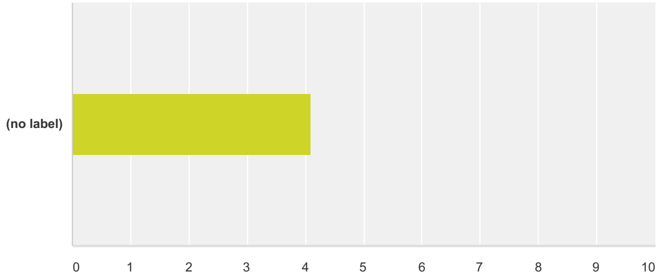


| | Strongly disagree | Disagree | Somewhat disagree | Somewhat agree | Agree | Strongly agree | Total | Weighted Average |
|---|---|---|---|---|---|---|---|---|
| (no label) | **8.18%** 9 | **11.82%** 13 | **15.45%** 17 | **21.82%** 24 | **11.82%** 13 | **30.91%** 34 | 110 | 4.10 |

## Q17 Imagine that in one year's time you will have a newborn baby who needs to be admitted to intensive care. At the same time, there will be another baby in need of treatment.One of the babies (Baby A) has a 100% chance of survival but will have a moderate learning and physical disability, while the other baby (Baby B) has a 100% chance of survival and will have a severe learning and physical disability.You don't know whether your baby will be Baby A or Baby B. Only one baby can be treated.However, you are able to vote now for a policy that will tell doctors what to do in situations like thisDo you:

Answered: 110 Skipped: 12

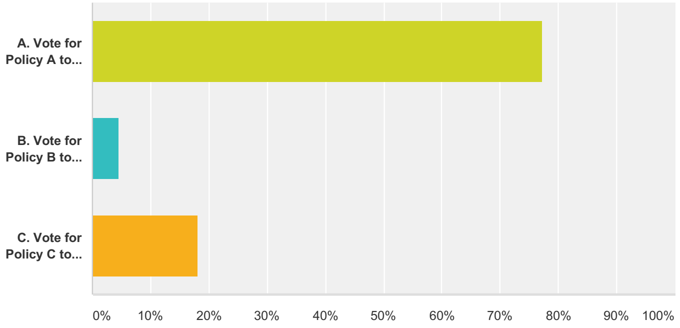


| Answer Choices | Responses | |
|---|---|---|
| A. Vote for Policy A to treat Baby A (moderate learning and physical disability) | **77.27%** | 85 |
| B. Vote for Policy B to treat Baby B (severe learning and physical disability) | **4.55%** | 5 |
| C. Vote for Policy C to toss a coin to decide which baby to treat | **18.18%** | 20 |
| **Total Respondents: 110** | | |

## Q18 Baby C has a 100% chance of survival and will be moderately disabled (refer to definition above).Baby D has a 100% chance of survival and will have no disability.Do you:

Answered: 110 Skipped: 12

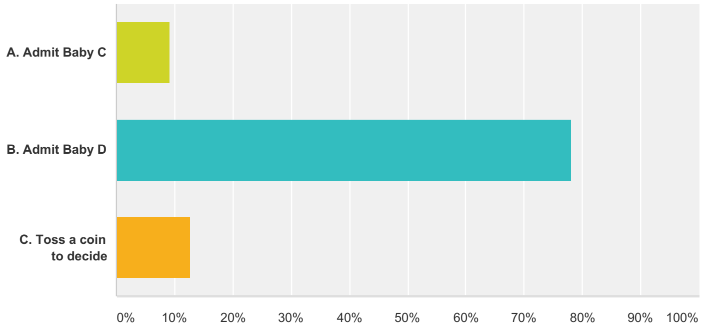


| Answer Choices | Responses | |
|---|---|---|
| A. Admit Baby C | **9.09%** | 10 |
| B. Admit Baby D | **78.18%** | 86 |
| C. Toss a coin to decide | **12.73%** | 14 |
| **Total Respondents: 110** | | |

## Q19 Baby E has a 100% chance of survival and will be mildly disabled.Baby F has a 100% chance of survival and will have no disability.Do you:

Answered: 110 Skipped: 12

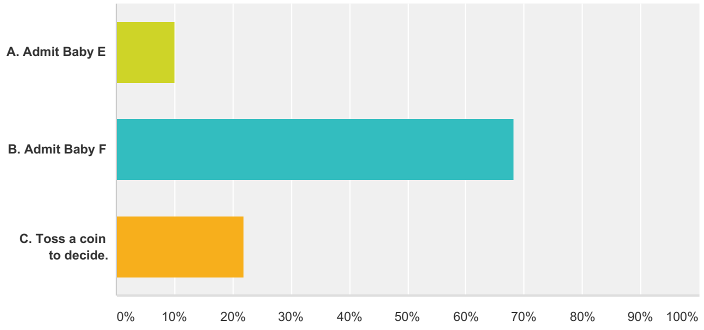


| Answer Choices | Responses | |
|---|---|---|
| A. Admit Baby E | **10.00%** | 11 |
| B. Admit Baby F | **68.18%** | 75 |
| C. Toss a coin to decide. | **21.82%** | 24 |
| **Total Respondents: 110** | | |

## Q20 Baby G has a 100% chance of survival and will be severely disabled.Baby H has a 100% chance of survival and will be mildly disabled.Do you:

Answered: 110 Skipped: 12

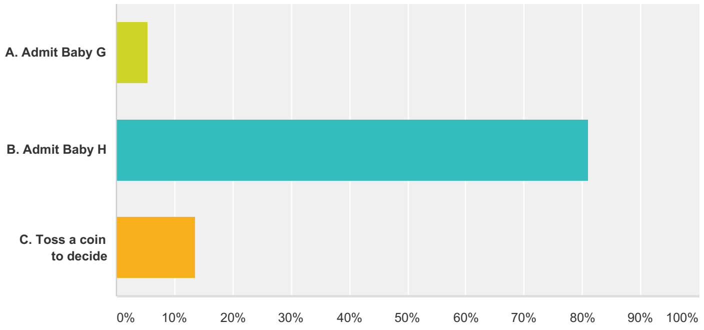


| Answer Choices | Responses | |
|---|---|---|
| A. Admit Baby G | **5.45%** | 6 |
| B. Admit Baby H | **80.91%** | 89 |
| C. Toss a coin to decide | **13.64%** | 15 |

| Total Respondents: 110 | |
|---|---|

## Q21 Baby J has a 100% chance of survival and will be moderately disabled.Baby K has a 100% chance of survival and will be severely disabled.Do you:

Answered: 110 Skipped: 12

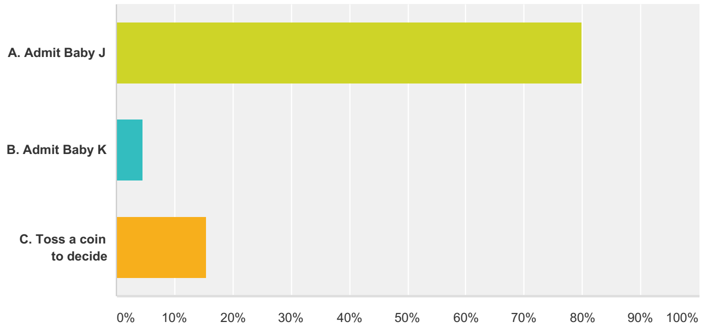


| Answer Choices | Responses | |
|---|---|---|
| A. Admit Baby J | 80.00% | 88 |
| B. Admit Baby K | 4.55% | 5 |
| C. Toss a coin to decide | 15.45% | 17 |
| Total Respondents: 110 | | |

## Q22 This newborn infant will survive the newborn period, but die at 15 years of age if they are admitted to the NICU. If a bed were available, would you admit this baby to the NICU?

Answered: 110 Skipped: 12

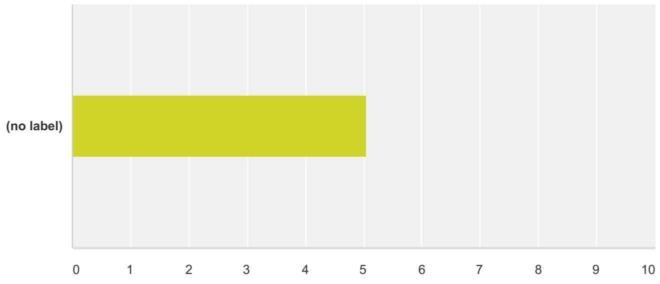


| | Strongly disagree | Disagree | Somewhat disagree | Somewhat agree | Agree | Strongly agree | Total | Weighted Average |
|---|---|---|---|---|---|---|---|---|
| (no label) | 2.73%<br>3 | 0.91%<br>1 | 1.82%<br>2 | 22.73%<br>25 | 26.36%<br>29 | 45.45%<br>50 | 110 | 5.05 |

## Q23 This newborn infant will survive the newborn period, but die at 5 years of age if they are admitted to the NICU. If a bed were available, would you admit this baby to the NICU?

Answered: 110 Skipped: 12

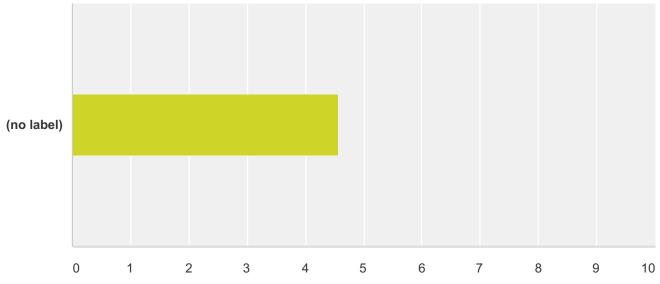


| | Strongly disagree | Disagree | Somewhat disagree | Somewhat agree | Agree | Strongly agree | Total | Weighted Average |
|---|---|---|---|---|---|---|---|---|

| | | | | | | | | |
|---|---|---|---|---|---|---|---|---|
| (no label) | **6.36%** 7 | **6.36%** 7 | **10.00%** 11 | **17.27%** 19 | **20.00%** 22 | **40.00%** 44 | 110 | 4.58 |

## Q24 This newborn infant will likely survive the newborn period, but die at 25 years of age if they are admitted to the NICU. If there were a bed available, would you admit this baby to the NICU?

Answered: 110 Skipped: 12

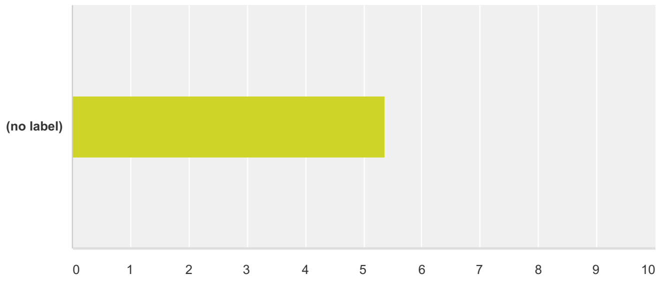


| | Strongly disagree | Disagree | Somewhat disagree | Somewhat agree | Agree | Strongly agree | Total | Weighted Average |
|---|---|---|---|---|---|---|---|---|
| (no label) | **1.82%** 2 | **0.00%** 0 | **0.00%** 0 | **13.64%** 15 | **26.36%** 29 | **58.18%** 64 | 110 | 5.37 |

## Q25 Baby A will survive with treatment and live to 15 years of age.Baby B will survive with treatment and live to normal adult life expectancy.Do you:

Answered: 110 Skipped: 12

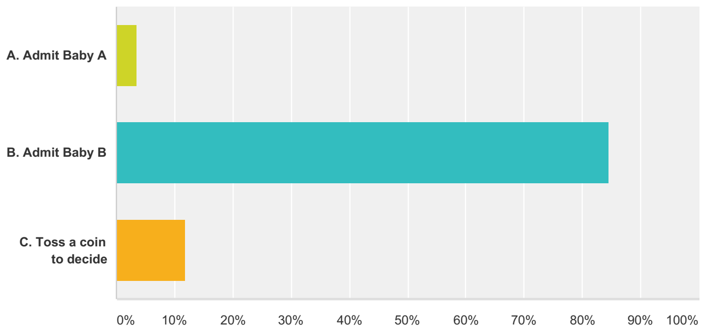


| Answer Choices | Responses | |
|---|---|---|
| A. Admit Baby A | **3.64%** | 4 |
| B. Admit Baby B | **84.55%** | 93 |
| C. Toss a coin to decide | **11.82%** | 13 |
| **Total Respondents: 110** | | |

## Q26 Baby C will survive with treatment and live to 5 years of age.Baby D will survive with treatment and live to 25 years of age.Do you:

Answered: 110 Skipped: 12

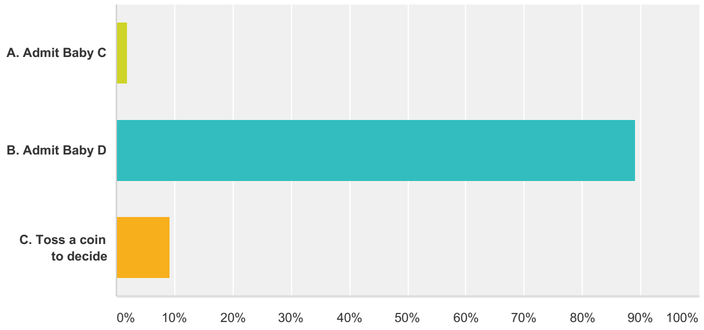

| Answer Choices | Responses | |
|---|---|---|
| A. Admit Baby C | 1.82% | 2 |
| B. Admit Baby D | 89.09% | 98 |
| C. Toss a coin to decide | 9.09% | 10 |
| Total Respondents: 110 | | |

## Q27 Baby E will survive with treatment and live to 15 years of age.Baby F will survive with treatment and live to 25 years of age.Do you:

Answered: 110 Skipped: 12

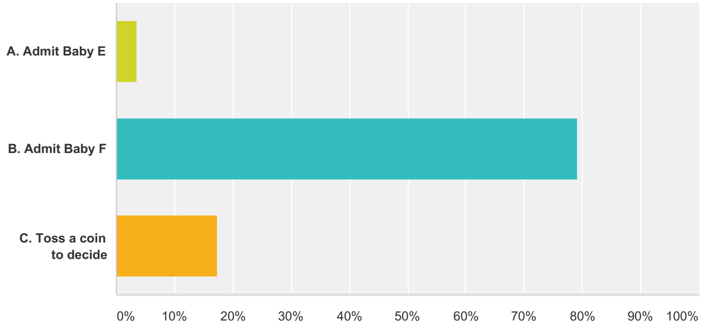


| Answer Choices | Responses | |
|---|---|---|
| A. Admit Baby E | 3.64% | 4 |
| B. Admit Baby F | 79.09% | 87 |
| C. Toss a coin to decide | 17.27% | 19 |
| Total Respondents: 110 | | |

## Q28 Baby G will survive with treatment and live to 41 years of age.Baby H will survive with treatment and live to 40 years of age.Do you:

Answered: 110 Skipped: 12

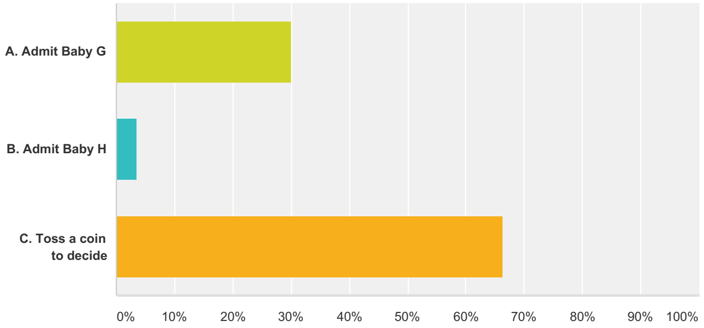


| Answer Choices | Responses | |
|---|---|---|
| A. Admit Baby G | 30.00% | 33 |
| B. Admit Baby H | 3.64% | 4 |
| C. Toss a coin to decide | 66.36% | 73 |
| Total Respondents: 110 | | |

## Q29 Baby J is expected to live to 5 years of age if not admitted to the NICU. If admitted, Baby J's life expectancy will increase to 10 years.Baby K is expected to live to 10 years of age if not admitted to the NICU. If admitted, Baby K's life expectancy will increase to 16 years.Do you:

Answered: 110 Skipped: 12

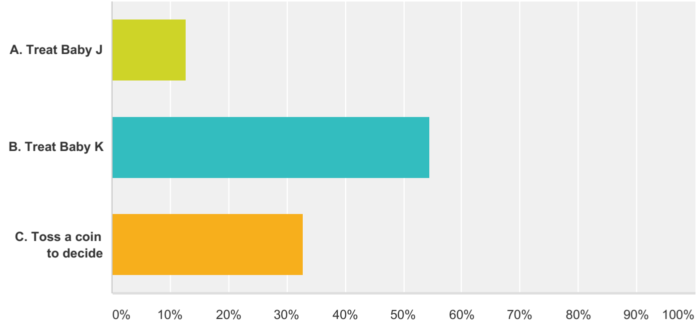


| Answer Choices | Responses | |
|---|---|---|
| A. Treat Baby J | 12.73% | 14 |
| B. Treat Baby K | 54.55% | 60 |
| C. Toss a coin to decide | 32.73% | 36 |
| Total Respondents: 110 | | |

## Q30 This newborn infant will survive the newborn period if treated, but this life-saving treatment will cost $200,000 USD. If there were a bed available, would you admit this baby to the NICU?

Answered: 109 Skipped: 13

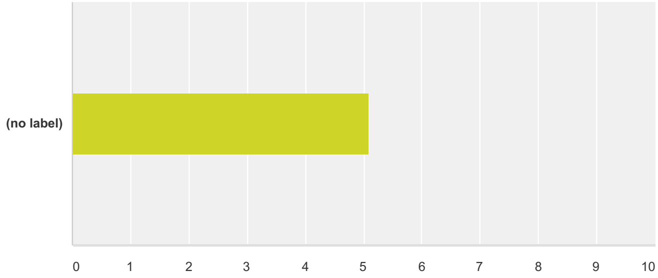


| | Strongly disagree | Disagree | Somewhat disagree | Somewhat agree | Agree | Strongly agree | Total | Weighted Average |
|---|---|---|---|---|---|---|---|---|
| (no label) | 2.75% 3 | 4.59% 5 | 0.92% 1 | 14.68% 16 | 26.61% 29 | 50.46% 55 | 109 | 5.09 |

## Q31 This newborn infant will survive the newborn period if treated, but this life-saving treatment will cost $7,000,000 (7 million) USD. If there were a bed available, would you admit this baby to the NICU?

Answered: 109 Skipped: 13

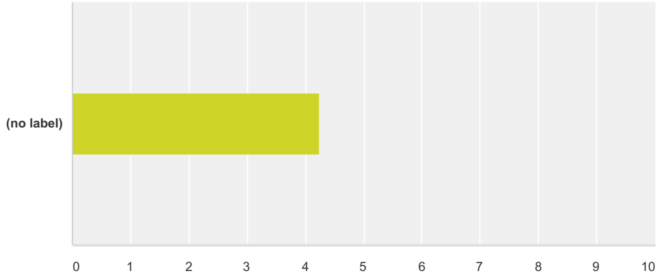


| | Strongly disagree | Disagree | Somewhat disagree | Somewhat agree | Agree | Strongly agree | Total | Weighted Average |
|---|---|---|---|---|---|---|---|---|
| (no label) | 9.17% 10 | 9.17% 10 | 11.01% 12 | 21.10% 23 | 19.27% 21 | 30.28% 33 | 109 | 4.23 |

## Q32 This newborn infant will survive the newborn period if treated, but this life-saving treatment will cost $10,000 USD. If there were a bed available, would you admit this baby to the NICU?

Answered: 109 Skipped: 13

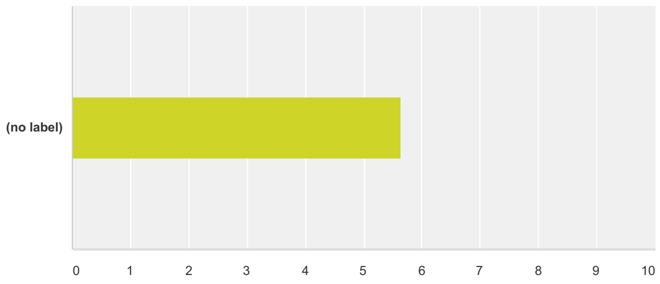


| | Strongly disagree | Disagree | Somewhat disagree | Somewhat agree | Agree | Strongly agree | Total | Weighted Average |
|---|---|---|---|---|---|---|---|---|
| (no label) | 0.00%<br>0 | 0.00%<br>0 | 0.92%<br>1 | 6.42%<br>7 | 20.18%<br>22 | 72.48%<br>79 | 109 | 5.64 |

## Q33 This newborn infant will survive the newborn period if treated, but this life-saving treatment will cost $150,000 USD. If there were a bed available, would you admit this baby to the NICU?

Answered: 109 Skipped: 13

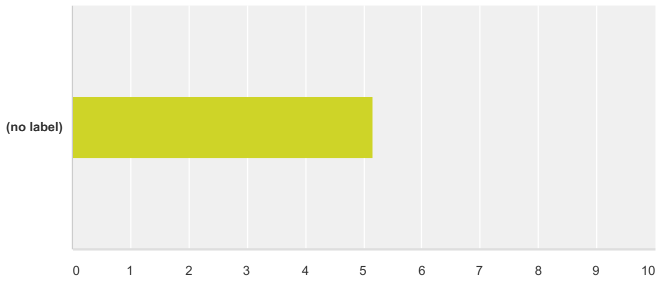


| | Strongly disagree | Disagree | Somewhat disagree | Somewhat agree | Agree | Strongly agree | Total | Weighted Average |
|---|---|---|---|---|---|---|---|---|
| (no label) | 0.92%<br>1 | 2.75%<br>3 | 4.59%<br>5 | 11.01%<br>12 | 33.03%<br>36 | 47.71%<br>52 | 109 | 5.16 |

## Q34 This newborn infant will survive the newborn period if treated, but this life-saving treatment will cost $5,000 USD. If there were a bed available, would you admit this baby to the NICU?

Answered: 109 Skipped: 13

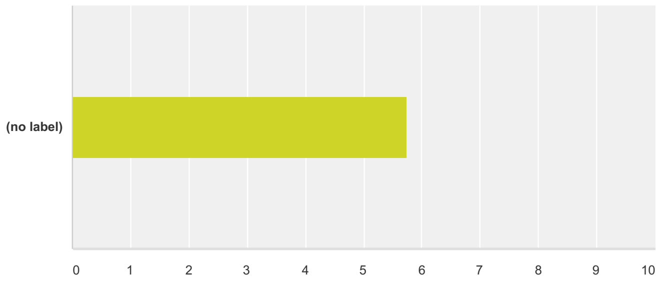


| | Strongly disagree | Disagree | Somewhat disagree | Somewhat agree | Agree | Strongly agree | Total | Weighted Average |
|---|---|---|---|---|---|---|---|---|
| (no label) | 0.00%<br>0 | 0.00%<br>0 | 0.00%<br>0 | 4.59%<br>5 | 15.60%<br>17 | 79.82%<br>87 | 109 | 5.75 |

## Q35 A life-saving treatment for Baby A costs $10,000 USD.A life-saving treatment for Baby B and Baby C costs $5,000 USD (each).You have a budget of $10,000 USD. Do you:

Answered: 109 Skipped: 13

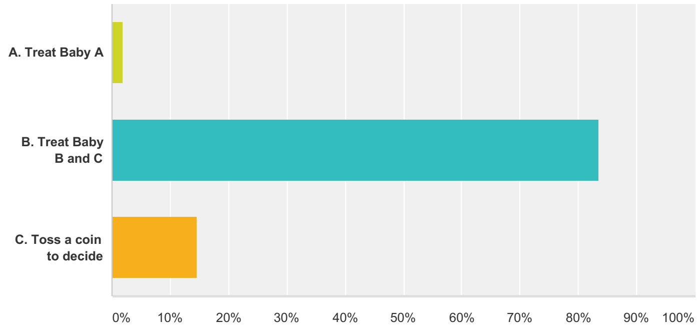


| Answer Choices | Responses | |
|---|---|---|
| A. Treat Baby A | 1.83% | 2 |
| B. Treat Baby B and C | 83.49% | 91 |
| C. Toss a coin to decide | 14.68% | 16 |
| Total Respondents: 109 | | |

## Q36 A life-saving treatment for Baby A costs $7,000,000 (7 million) USD.A life-saving treatment for 70 babies costs $100,000 USD (each).You have a budget of $7,000,000 (7 million) USD. Do you:

Answered: 109 Skipped: 13

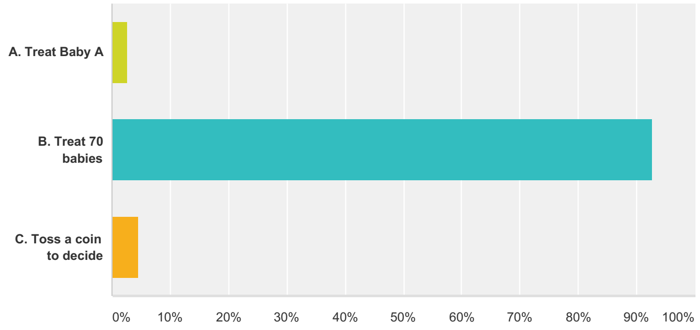


| Answer Choices | Responses | |
|---|---|---|
| A. Treat Baby A | 2.75% | 3 |
| B. Treat 70 babies | 92.66% | 101 |
| C. Toss a coin to decide | 4.59% | 5 |
| Total Respondents: 109 | | |

## Q37 A life-saving treatment for Baby A costs $150,000 USD.A life-saving treatment for Baby B, C, D, E, F, G, H, I, J and K costs $15,000 USD (each).You have a budget of $150,000 USD. Do you:

Answered: 109 Skipped: 13

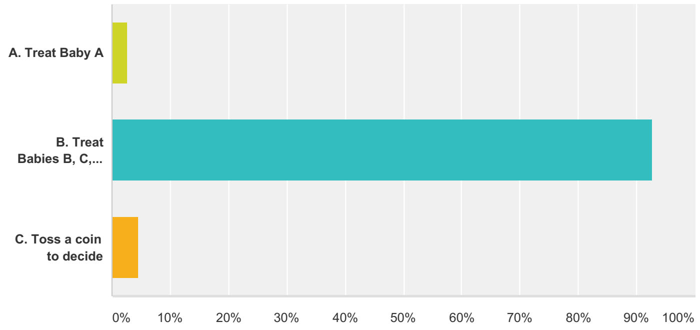


| Answer Choices | Responses | |
|---|---|---|
| A. Treat Baby A | 2.75% | 3 |

| | | |
|---|---|---|
| B. Treat Babies B, C, D, E, F, G, H, I, J and K | **92.66%** | 101 |
| C. Toss a coin to decide | **4.59%** | 5 |
| **Total Respondents: 109** | | |

## Q38 A life-saving treatment for Baby A costs $1,000 USD.A life-saving treatment for Baby B, C, D, E and F costs $200 USD (each).You have a budget of $1000 USD. Do you:

Answered: 109 Skipped: 13

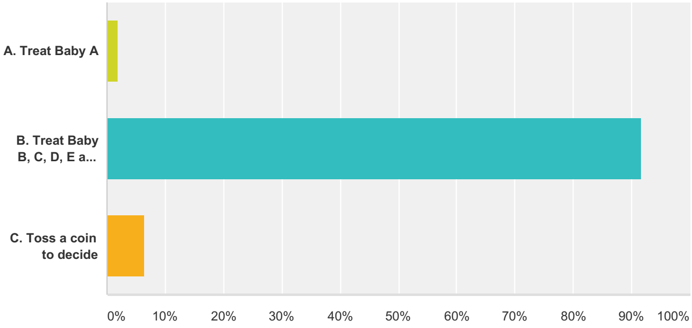


| Answer Choices | Responses | |
|---|---|---|
| A. Treat Baby A | **1.83%** | 2 |
| B. Treat Baby B, C, D, E and F | **91.74%** | 100 |
| C. Toss a coin to decide | **6.42%** | 7 |
| **Total Respondents: 109** | | |

## Q39 What year were you born?

Answered: 109 Skipped: 13

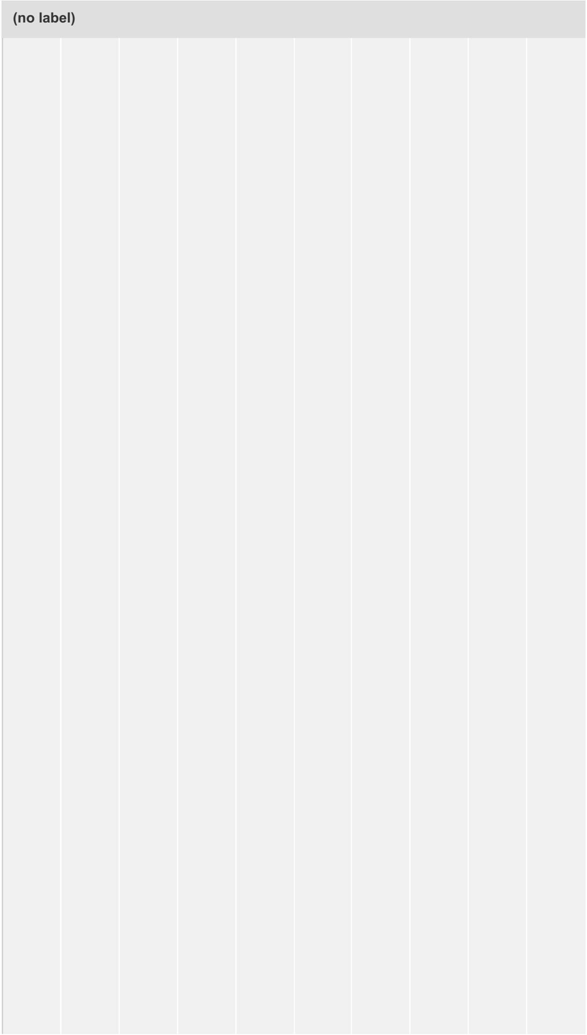

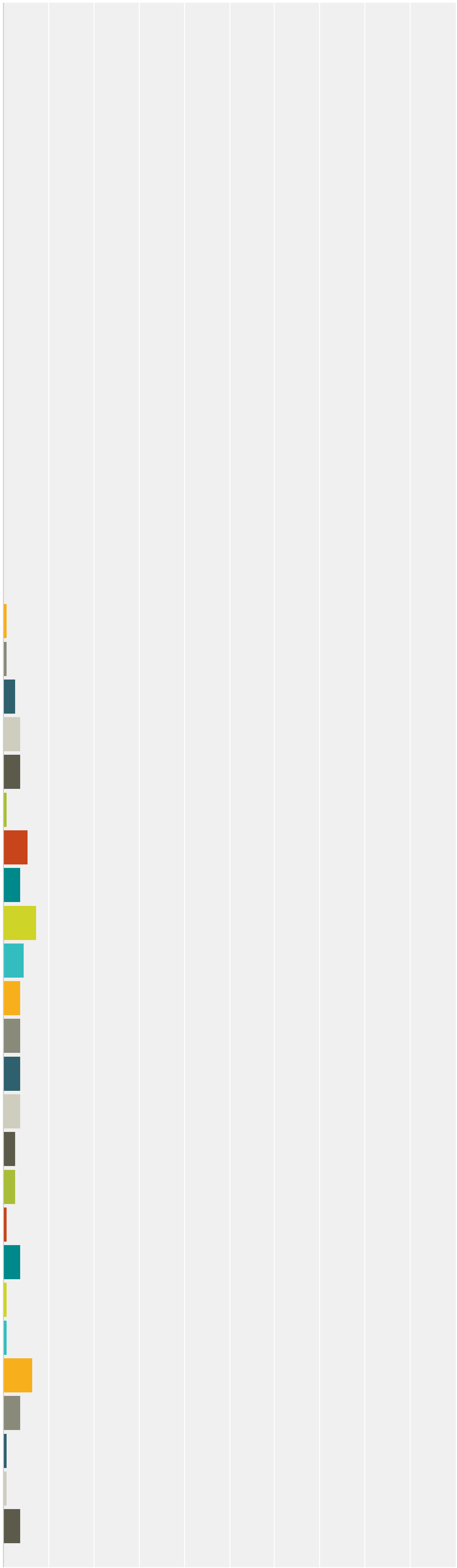

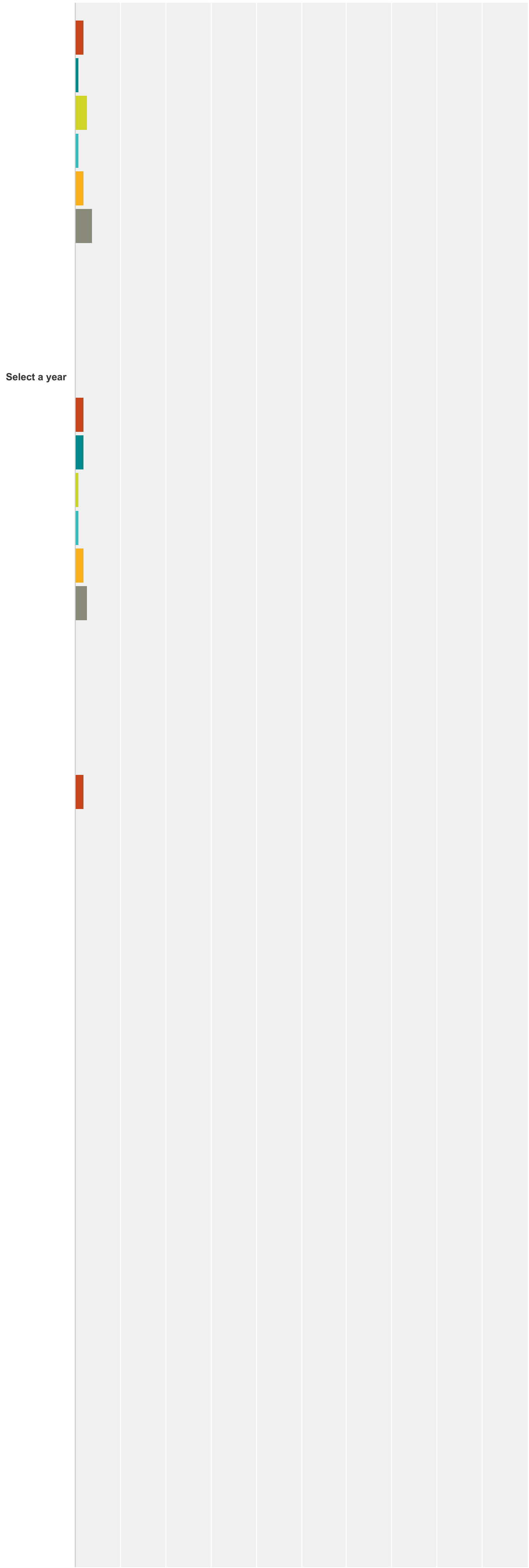
Select a year

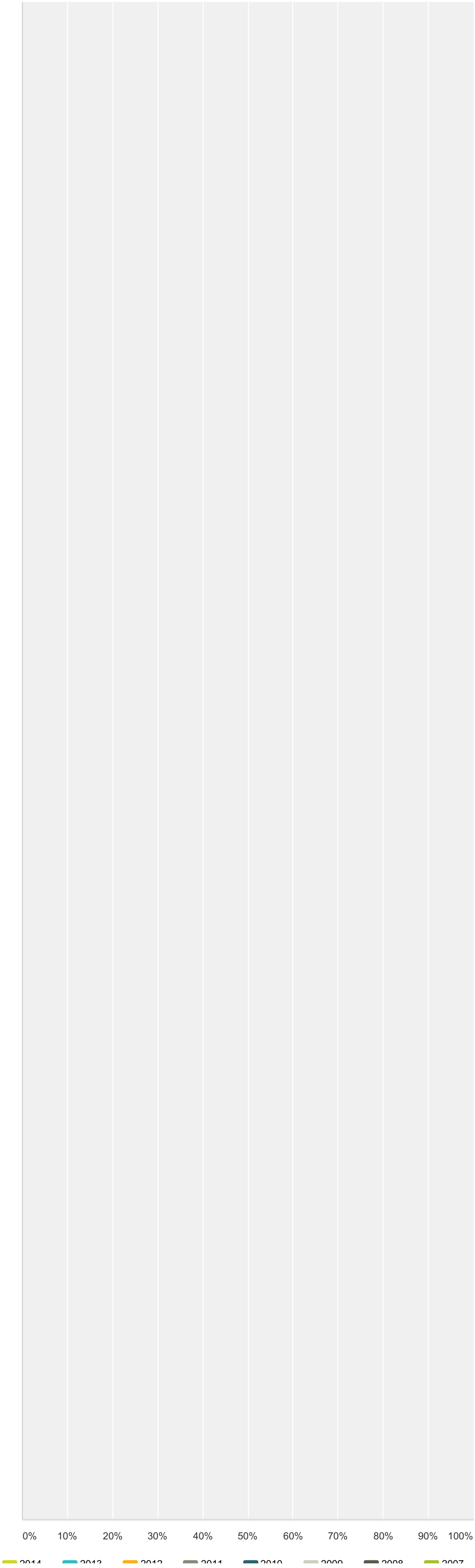
0%
10%
20%
30%
40%
50%
60%
70%
80%
90%
100%

2014 2013 2012 2011 2010 2009 2008 2007
2006 2005 2004 2003 2002 2001 2000 1999
1998 1997 1996 1995 1994 1993 1992 1991
1990 1989 1988 1987 1986 1985 1984 1983
1982 1981 1980 1979 1978 1977 1976 1975
1974 1973 1972 1971 1970 1969 1968 1967
1966 1965 1964 1963 1962 1961 1960 1959
1958 1957 1956 1955 1954 1953 1952 1951
1950 1949 1948 1947 1946 1945 1944 1943
1942 1941 1940 1939 1938 1937 1936 1935
1934 1933 1932 1931 1930 1929 1928 1927
1926 1925 1924 1923 1922 1921 1920 1919
1918 1917 1916 1915 1914 1913 1912 1911
1910 1909 1908 1907 1906 1905 1904 1903
1902 1901 1900

(no label)

| | 2014 | 2013 | 2012 | 2011 | 2010 | 2009 | 2008 | 2007 | 2006 | 2005 | 2004 | 2003 | 2002 | 2001 | 2000 | 1999 | 1998 | 1997 | 1996 | 1995 | 1994 | 1993 | 1[illegible] |
|---|---|---|---|---|---|---|---|---|---|---|---|---|---|---|---|---|---|---|---|---|---|---|---|
| Select a year | 0.00%<br>0 | 0.00%<br>0 | 0.00%<br>0 | 0.00%<br>0 | 0.00%<br>0 | 0.00%<br>0 | 0.00%<br>0 | 0.00%<br>0 | 0.00%<br>0 | 0.00%<br>0 | 0.00%<br>0 | 0.00%<br>0 | 0.00%<br>0 | 0.00%<br>0 | 0.00%<br>0 | 0.00%<br>0 | 0.00%<br>0 | 0.00%<br>0 | 0.00%<br>0 | 0.00%<br>0 | 0.00%<br>0 | 0.00%<br>0 | 0.[illegible] |

## Q40 What is your gender?

Answered: 109 Skipped: 13

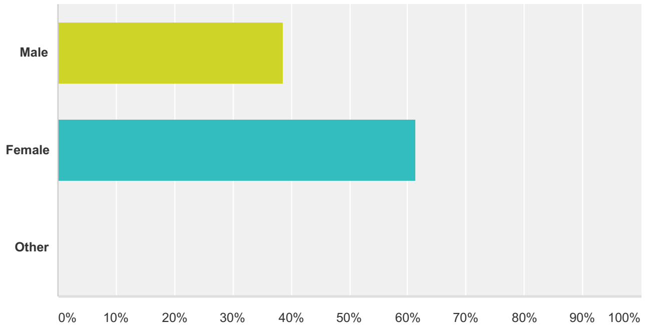


| Answer Choices | Responses | |
|---|---|---|
| Male | 38.53% | 42 |
| Female | 61.47% | 67 |
| Other | 0.00% | 0 |
| Total Respondents: 109 | | |

## Q41 Are you a parent?

Answered: 109 Skipped: 13

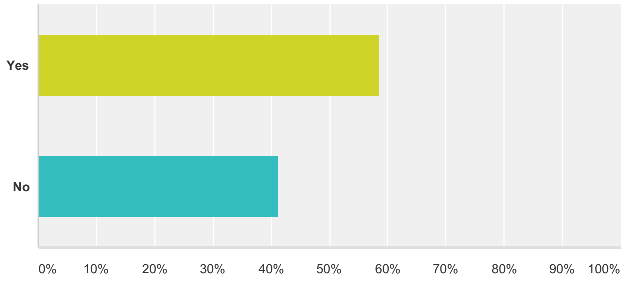


| Answer Choices | Responses | |
|---|---|---|
| Yes | 58.72% | 64 |
| No | 41.28% | 45 |
| Total Respondents: 109 | | |

## Q42 What is your nationality?

Answered: 109 Skipped: 13

| # | Responses | Date |
|---|---|---|
| 1 | American | 8/12/2014 2:43 AM |
| 2 | American | 8/12/2014 2:43 AM |

| | | |
|---|---|---|
| 3 | Indian | 8/11/2014 2:07 PM |
| 4 | American | 8/7/2014 11:12 AM |
| 5 | American | 8/7/2014 6:41 AM |
| 6 | American | 8/6/2014 5:01 PM |
| 7 | American | 8/6/2014 5:25 AM |
| 8 | American | 8/6/2014 4:22 AM |
| 9 | American | 8/6/2014 2:42 AM |
| 10 | native american | 8/5/2014 3:56 PM |
| 11 | American | 8/5/2014 6:28 AM |
| 12 | American | 8/5/2014 12:07 AM |
| 13 | American | 8/4/2014 10:57 AM |
| 14 | American | 8/4/2014 10:31 AM |
| 15 | Indian | 8/4/2014 10:07 AM |
| 16 | American | 8/3/2014 11:33 AM |
| 17 | indian | 8/3/2014 5:29 AM |
| 18 | White | 8/2/2014 11:57 AM |
| 19 | American | 8/2/2014 5:32 AM |
| 20 | American | 8/1/2014 7:22 PM |
| 21 | american | 8/1/2014 4:13 AM |
| 22 | American | 7/31/2014 9:03 PM |
| 23 | American | 7/31/2014 1:46 PM |
| 24 | American | 7/31/2014 1:14 PM |
| 25 | American | 7/30/2014 5:31 PM |
| 26 | USA | 7/29/2014 2:30 PM |
| 27 | American | 7/29/2014 6:30 AM |
| 28 | Indian | 7/29/2014 3:28 AM |
| 29 | Caucasian | 7/29/2014 2:35 AM |
| 30 | American | 7/28/2014 8:50 AM |
| 31 | Indian | 7/27/2014 3:59 AM |
| 32 | Indian | 7/27/2014 3:44 AM |
| 33 | American | 7/23/2014 2:58 PM |
| 34 | American | 7/22/2014 9:25 AM |
| 35 | United States | 7/22/2014 6:44 AM |
| 36 | white | 7/22/2014 1:37 AM |
| 37 | American | 7/21/2014 11:47 PM |
| 38 | american | 7/21/2014 1:57 PM |
| 39 | American | 7/21/2014 4:21 AM |
| 40 | american | 7/20/2014 7:40 AM |
| 41 | american | 7/20/2014 7:27 AM |
| 42 | Indian | 7/19/2014 11:13 PM |
| 43 | American | 7/18/2014 1:34 AM |
| 44 | filipino | 7/17/2014 8:09 PM |
| 45 | Indian | 7/15/2014 11:38 PM |
| 46 | USA | 7/15/2014 6:16 PM |
| 47 | usa | 7/15/2014 1:22 PM |
| 48 | usa | 7/15/2014 11:39 AM |
| 49 | American | 7/15/2014 8:39 AM |
| 50 | European American | 7/15/2014 5:53 AM |
| 51 | Indian | 7/15/2014 4:36 AM |
| 52 | USA | 7/15/2014 4:26 AM |
| 53 | American | 7/15/2014 2:07 AM |
| 54 | American | 7/15/2014 1:51 AM |
| 55 | american | 7/15/2014 12:36 AM |
| 56 | Indian | 7/14/2014 11:41 PM |
| 57 | American | 7/14/2014 11:20 PM |
| 58 | United States | 7/14/2014 7:52 PM |
| 59 | american | 7/14/2014 3:24 PM |
| 60 | USA | 7/14/2014 9:58 AM |
| 61 | white | 7/14/2014 5:31 AM |
| 62 | American | 7/14/2014 2:17 AM |
| 63 | American | 7/14/2014 1:51 AM |
| 64 | American | 7/14/2014 1:26 AM |
| 65 | Indian | 7/13/2014 8:41 PM |
| 66 | American | 7/13/2014 4:30 PM |
| 67 | usa | 7/13/2014 1:54 PM |

| | | |
|---|---|---|
| 68 | american | 7/13/2014 1:46 PM |
| 69 | usa | 7/13/2014 1:37 PM |
| 70 | usa | 7/13/2014 12:53 PM |
| 71 | United States | 7/13/2014 9:20 AM |
| 72 | usa | 7/13/2014 9:08 AM |
| 73 | American | 7/13/2014 8:37 AM |
| 74 | american | 7/13/2014 7:45 AM |
| 75 | American | 7/13/2014 7:32 AM |
| 76 | American | 7/13/2014 7:18 AM |
| 77 | White | 7/13/2014 7:14 AM |
| 78 | usa | 7/13/2014 7:07 AM |
| 79 | American | 7/13/2014 5:49 AM |
| 80 | USA | 7/13/2014 5:23 AM |
| 81 | American | 7/13/2014 5:23 AM |
| 82 | American | 7/13/2014 4:19 AM |
| 83 | American | 7/13/2014 4:18 AM |
| 84 | American | 7/13/2014 3:51 AM |
| 85 | USA | 7/13/2014 3:46 AM |
| 86 | American | 7/13/2014 3:46 AM |
| 87 | American | 7/13/2014 3:44 AM |
| 88 | American | 7/13/2014 2:41 AM |
| 89 | USA | 7/13/2014 2:39 AM |
| 90 | American | 7/13/2014 2:33 AM |
| 91 | usa | 7/13/2014 2:20 AM |
| 92 | American | 7/13/2014 2:11 AM |
| 93 | American | 7/13/2014 1:59 AM |
| 94 | British | 7/13/2014 1:51 AM |
| 95 | American | 7/13/2014 1:30 AM |
| 96 | Yes | 7/13/2014 1:25 AM |
| 97 | usa | 7/13/2014 1:23 AM |
| 98 | Caucasian | 7/13/2014 1:00 AM |
| 99 | USA | 7/13/2014 12:57 AM |
| 100 | American | 7/13/2014 12:57 AM |
| 101 | german | 7/13/2014 12:54 AM |
| 102 | Indian | 7/13/2014 12:47 AM |
| 103 | American | 7/13/2014 12:36 AM |
| 104 | Indian | 7/13/2014 12:32 AM |
| 105 | Indian | 7/12/2014 11:26 PM |
| 106 | Black American | 7/12/2014 11:25 PM |
| 107 | american | 7/12/2014 10:44 PM |
| 108 | Indian | 7/12/2014 9:40 PM |
| 109 | American | 7/12/2014 9:23 PM |

## Q43 What country do you live in?

**Answered: 109 Skipped: 13**

| # | Responses | Date |
|---|---|---|
| 1 | USA | 8/12/2014 2:43 AM |
| 2 | US | 8/12/2014 2:43 AM |
| 3 | India | 8/11/2014 2:07 PM |
| 4 | USA | 8/7/2014 11:12 AM |
| 5 | USA | 8/7/2014 6:41 AM |
| 6 | USA | 8/6/2014 5:01 PM |
| 7 | United States | 8/6/2014 5:25 AM |
| 8 | The U.S.A. | 8/6/2014 4:22 AM |
| 9 | USA | 8/6/2014 2:42 AM |
| 10 | united states | 8/5/2014 3:56 PM |
| 11 | USA | 8/5/2014 6:28 AM |
| 12 | USA | 8/5/2014 12:07 AM |
| 13 | USA | 8/4/2014 10:57 AM |
| 14 | USA | 8/4/2014 10:31 AM |
| 15 | United States | 8/4/2014 10:07 AM |
| 16 | USA | 8/3/2014 11:33 AM |
| 17 | india | 8/3/2014 5:29 AM |
| 18 | United States | 8/2/2014 11:57 AM |

| | | |
|---|---|---|
| 19 | USA | 8/2/2014 5:32 AM |
| 20 | USA | 8/1/2014 7:22 PM |
| 21 | us | 8/1/2014 4:13 AM |
| 22 | United States | 7/31/2014 9:03 PM |
| 23 | USA | 7/31/2014 1:46 PM |
| 24 | United States | 7/31/2014 1:14 PM |
| 25 | USA | 7/30/2014 5:31 PM |
| 26 | USA | 7/29/2014 2:30 PM |
| 27 | United States | 7/29/2014 6:30 AM |
| 28 | India | 7/29/2014 3:28 AM |
| 29 | USA | 7/29/2014 2:35 AM |
| 30 | US | 7/28/2014 8:50 AM |
| 31 | India | 7/27/2014 3:59 AM |
| 32 | India | 7/27/2014 3:44 AM |
| 33 | United States | 7/23/2014 2:58 PM |
| 34 | United States | 7/22/2014 9:25 AM |
| 35 | United States | 7/22/2014 6:44 AM |
| 36 | USA | 7/22/2014 1:37 AM |
| 37 | USA | 7/21/2014 11:47 PM |
| 38 | usa | 7/21/2014 1:57 PM |
| 39 | USA | 7/21/2014 4:21 AM |
| 40 | USA | 7/20/2014 7:40 AM |
| 41 | USA | 7/20/2014 7:27 AM |
| 42 | India | 7/19/2014 11:13 PM |
| 43 | USA | 7/18/2014 1:34 AM |
| 44 | USA | 7/17/2014 8:09 PM |
| 45 | India | 7/15/2014 11:38 PM |
| 46 | USA | 7/15/2014 6:16 PM |
| 47 | usa | 7/15/2014 1:22 PM |
| 48 | usa | 7/15/2014 11:39 AM |
| 49 | USA | 7/15/2014 8:39 AM |
| 50 | USA | 7/15/2014 5:53 AM |
| 51 | India | 7/15/2014 4:36 AM |
| 52 | USA | 7/15/2014 4:26 AM |
| 53 | USA | 7/15/2014 2:07 AM |
| 54 | USA | 7/15/2014 1:51 AM |
| 55 | united states | 7/15/2014 12:36 AM |
| 56 | India | 7/14/2014 11:41 PM |
| 57 | United States | 7/14/2014 11:20 PM |
| 58 | United States | 7/14/2014 7:52 PM |
| 59 | usa | 7/14/2014 3:24 PM |
| 60 | USA | 7/14/2014 9:58 AM |
| 61 | USA | 7/14/2014 5:31 AM |
| 62 | USA | 7/14/2014 2:17 AM |
| 63 | United States | 7/14/2014 1:51 AM |
| 64 | USA | 7/14/2014 1:26 AM |
| 65 | India | 7/13/2014 8:41 PM |
| 66 | USA | 7/13/2014 4:30 PM |
| 67 | usa | 7/13/2014 1:54 PM |
| 68 | usa | 7/13/2014 1:46 PM |
| 69 | usa | 7/13/2014 1:37 PM |
| 70 | usa | 7/13/2014 12:53 PM |
| 71 | United States | 7/13/2014 9:20 AM |
| 72 | usa | 7/13/2014 9:08 AM |
| 73 | USA | 7/13/2014 8:37 AM |
| 74 | USA | 7/13/2014 7:45 AM |
| 75 | USA | 7/13/2014 7:32 AM |
| 76 | USA | 7/13/2014 7:18 AM |
| 77 | United States | 7/13/2014 7:14 AM |
| 78 | usa | 7/13/2014 7:07 AM |
| 79 | United States of America | 7/13/2014 5:49 AM |
| 80 | USA | 7/13/2014 5:23 AM |
| 81 | The United States of America | 7/13/2014 5:23 AM |
| 82 | USA | 7/13/2014 4:19 AM |
| 83 | United States | 7/13/2014 4:18 AM |

| | | |
|---|---|---|
| 84 | United States | 7/13/2014 3:51 AM |
| 85 | USA | 7/13/2014 3:46 AM |
| 86 | USA | 7/13/2014 3:46 AM |
| 87 | United States | 7/13/2014 3:44 AM |
| 88 | United States | 7/13/2014 2:41 AM |
| 89 | USA | 7/13/2014 2:39 AM |
| 90 | USA | 7/13/2014 2:33 AM |
| 91 | usa | 7/13/2014 2:20 AM |
| 92 | United States | 7/13/2014 2:11 AM |
| 93 | United States | 7/13/2014 1:59 AM |
| 94 | US | 7/13/2014 1:51 AM |
| 95 | USA | 7/13/2014 1:30 AM |
| 96 | USA | 7/13/2014 1:25 AM |
| 97 | usa | 7/13/2014 1:23 AM |
| 98 | USA | 7/13/2014 1:00 AM |
| 99 | USA | 7/13/2014 12:57 AM |
| 100 | USA | 7/13/2014 12:57 AM |
| 101 | united states | 7/13/2014 12:54 AM |
| 102 | India | 7/13/2014 12:47 AM |
| 103 | USA | 7/13/2014 12:36 AM |
| 104 | India | 7/13/2014 12:32 AM |
| 105 | India | 7/12/2014 11:26 PM |
| 106 | United States | 7/12/2014 11:25 PM |
| 107 | USA | 7/12/2014 10:44 PM |
| 108 | India | 7/12/2014 9:40 PM |
| 109 | USA | 7/12/2014 9:23 PM |

## Q44 Are you religious?

Answered: 109 Skipped: 13

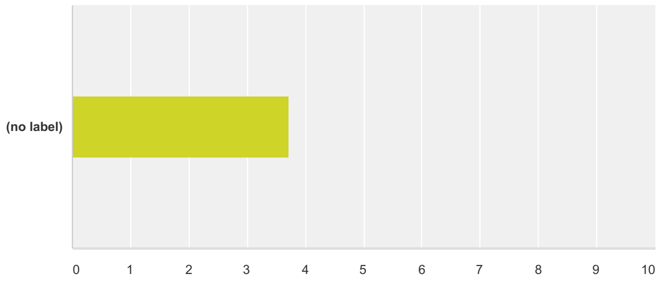


| | Strongly Disagree | Disagree | Somewhat Disagree | Neutral | Somewhat Agree | Agree | Strongly Agree | Total | Weighted Average |
|---|---|---|---|---|---|---|---|---|---|
| (no label) | **32.11%** 35 | **10.09%** 11 | **2.75%** 3 | **7.34%** 8 | **17.43%** 19 | **17.43%** 19 | **12.84%** 14 | 109 | 3.72 |

## Q45 What is your religion?

Answered: 109 Skipped: 13

| # | Responses | Date |
|---|---|---|
| 1 | Protestant | 8/12/2014 2:43 AM |
| 2 | Jewish | 8/12/2014 2:43 AM |
| 3 | Islam | 8/11/2014 2:07 PM |
| 4 | Atheist | 8/7/2014 11:12 AM |
| 5 | None | 8/7/2014 6:41 AM |
| 6 | none | 8/6/2014 5:01 PM |
| 7 | Roman Catholic | 8/6/2014 5:25 AM |
| 8 | None | 8/6/2014 4:22 AM |
| 9 | None | 8/6/2014 2:42 AM |
| 10 | baptist | 8/5/2014 3:56 PM |
| 11 | agnostic | 8/5/2014 6:28 AM |
| 12 | atheist | 8/5/2014 12:07 AM |
| 13 | Christian | 8/4/2014 10:57 AM |
| 14 | I have no religious affiliation. | 8/4/2014 10:31 AM |
| 15 | Hindu | 8/4/2014 10:07 AM |
| 16 | N/A | 8/3/2014 11:33 AM |

| | | |
|---|---|---|
| 17 | muslim | 8/3/2014 5:29 AM |
| 18 | Agnostic | 8/2/2014 11:57 AM |
| 19 | Christianity | 8/2/2014 5:32 AM |
| 20 | Lutheran | 8/1/2014 7:22 PM |
| 21 | none | 8/1/2014 4:13 AM |
| 22 | Christianity | 7/31/2014 9:03 PM |
| 23 | Agnostic | 7/31/2014 1:46 PM |
| 24 | Agnostic | 7/31/2014 1:14 PM |
| 25 | N/A | 7/30/2014 5:31 PM |
| 26 | none | 7/29/2014 2:30 PM |
| 27 | Pagan | 7/29/2014 6:30 AM |
| 28 | Hindu | 7/29/2014 3:28 AM |
| 29 | Athiest | 7/29/2014 2:35 AM |
| 30 | Lutheran | 7/28/2014 8:50 AM |
| 31 | Hindu | 7/27/2014 3:59 AM |
| 32 | Hindu | 7/27/2014 3:44 AM |
| 33 | None | 7/23/2014 2:58 PM |
| 34 | agnostic | 7/22/2014 9:25 AM |
| 35 | atheist | 7/22/2014 6:44 AM |
| 36 | Pentecostal Christian | 7/22/2014 1:37 AM |
| 37 | none | 7/21/2014 11:47 PM |
| 38 | protestant | 7/21/2014 1:57 PM |
| 39 | Christian | 7/21/2014 4:21 AM |
| 40 | Catholic | 7/20/2014 7:40 AM |
| 41 | Buddhism | 7/20/2014 7:27 AM |
| 42 | Islam | 7/19/2014 11:13 PM |
| 43 | Christian - Methodist | 7/18/2014 1:34 AM |
| 44 | catholic | 7/17/2014 8:09 PM |
| 45 | Hindu | 7/15/2014 11:38 PM |
| 46 | Spiritual | 7/15/2014 6:16 PM |
| 47 | non-denominational christian | 7/15/2014 1:22 PM |
| 48 | spiritual, not religious | 7/15/2014 11:39 AM |
| 49 | Christian | 7/15/2014 8:39 AM |
| 50 | Seventh Day Adventist | 7/15/2014 5:53 AM |
| 51 | Hindhu | 7/15/2014 4:36 AM |
| 52 | Christian | 7/15/2014 4:26 AM |
| 53 | Christian | 7/15/2014 2:07 AM |
| 54 | Buddhism | 7/15/2014 1:51 AM |
| 55 | none | 7/15/2014 12:36 AM |
| 56 | Hindu | 7/14/2014 11:41 PM |
| 57 | no affiliation | 7/14/2014 11:20 PM |
| 58 | Agnostic | 7/14/2014 7:52 PM |
| 59 | christian | 7/14/2014 3:24 PM |
| 60 | Christianity - Protestant | 7/14/2014 9:58 AM |
| 61 | catholic | 7/14/2014 5:31 AM |
| 62 | Athiest | 7/14/2014 2:17 AM |
| 63 | Baptist | 7/14/2014 1:51 AM |
| 64 | ROman Catholic | 7/14/2014 1:26 AM |
| 65 | Hindu | 7/13/2014 8:41 PM |
| 66 | Christian | 7/13/2014 4:30 PM |
| 67 | none | 7/13/2014 1:54 PM |
| 68 | none | 7/13/2014 1:46 PM |
| 69 | Christian | 7/13/2014 1:37 PM |
| 70 | judaism | 7/13/2014 12:53 PM |
| 71 | none | 7/13/2014 9:20 AM |
| 72 | christian | 7/13/2014 9:08 AM |
| 73 | Buddhist | 7/13/2014 8:37 AM |
| 74 | no particular religion | 7/13/2014 7:45 AM |
| 75 | Atheist | 7/13/2014 7:32 AM |
| 76 | none | 7/13/2014 7:18 AM |
| 77 | Spiritual | 7/13/2014 7:14 AM |
| 78 | judaism | 7/13/2014 7:07 AM |
| 79 | Christian | 7/13/2014 5:49 AM |
| 80 | CHRISTIAN | 7/13/2014 5:23 AM |
| 81 | None | 7/13/2014 5:23 AM |

| | | |
|---|---|---|
| 82 | n/a | 7/13/2014 4:19 AM |
| 83 | Episcopalian | 7/13/2014 4:18 AM |
| 84 | Unaffiliated | 7/13/2014 3:51 AM |
| 85 | n/a | 7/13/2014 3:46 AM |
| 86 | Christian | 7/13/2014 3:46 AM |
| 87 | Catholic | 7/13/2014 3:44 AM |
| 88 | Atheist | 7/13/2014 2:41 AM |
| 89 | Roman Catholic | 7/13/2014 2:39 AM |
| 90 | NONE | 7/13/2014 2:33 AM |
| 91 | none | 7/13/2014 2:20 AM |
| 92 | N/A | 7/13/2014 2:11 AM |
| 93 | atheist | 7/13/2014 1:59 AM |
| 94 | atheist | 7/13/2014 1:51 AM |
| 95 | Evangelical Christian | 7/13/2014 1:30 AM |
| 96 | None | 7/13/2014 1:25 AM |
| 97 | catholic | 7/13/2014 1:23 AM |
| 98 | Agnostic | 7/13/2014 1:00 AM |
| 99 | Spiritual | 7/13/2014 12:57 AM |
| 100 | Agnostic | 7/13/2014 12:57 AM |
| 101 | christain | 7/13/2014 12:54 AM |
| 102 | Hindu | 7/13/2014 12:47 AM |
| 103 | Agnostic | 7/13/2014 12:36 AM |
| 104 | Hindu | 7/13/2014 12:32 AM |
| 105 | Hindu | 7/12/2014 11:26 PM |
| 106 | Christian | 7/12/2014 11:25 PM |
| 107 | baptist | 7/12/2014 10:44 PM |
| 108 | Christianity | 7/12/2014 9:40 PM |
| 109 | Roman Catholic | 7/12/2014 9:23 PM |

## Q46 What is your marital status?

Answered: 109 Skipped: 13

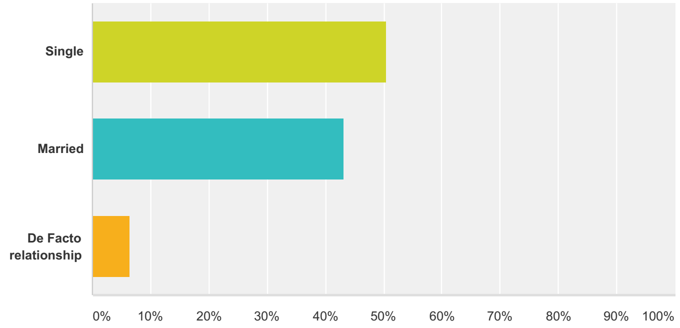


| Answer Choices | Responses | |
|---|---|---|
| Single | **50.46%** | 55 |
| Married | **43.12%** | 47 |
| De Facto relationship | **6.42%** | 7 |
| **Total Respondents: 109** | | |

## Q47 What is your highest level of education?

Answered: 109 Skipped: 13

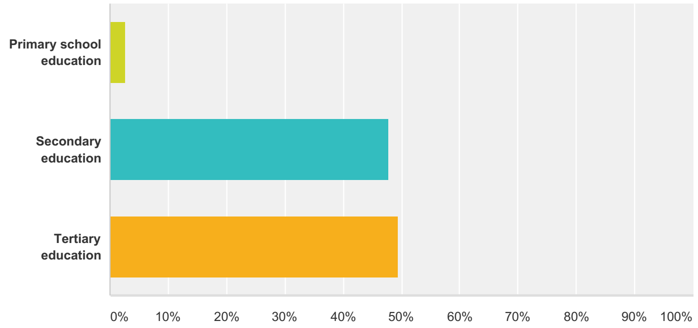

| Answer Choices | Responses | |
|---|---|---|
| Primary school education | 2.75% | 3 |
| Secondary education | 47.71% | 52 |
| Tertiary education | 49.54% | 54 |
| Total Respondents: 109 | | |

## Q48 Imagine you are manning the sole coastguard boat on duty. Two boats have been overturned some distance from each other. There are 5 people on one life raft 50 miles due north, and 1 person on another life raft 50 miles due south. A storm is brewing and it is highly likely you will only be able to reach one life raft before the storm overturns them and the people drown.Do you:

Answered: 109 Skipped: 13

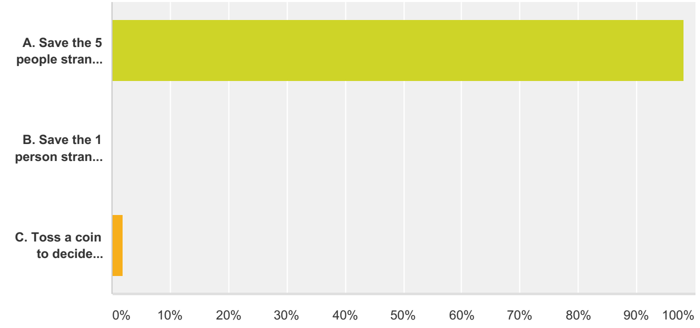


| Answer Choices | Responses | |
|---|---|---|
| A. Save the 5 people stranded on the life raft 50 miles due north | 98.17% | 107 |
| B. Save the 1 person stranded on the life raft 50 miles due south | 0.00% | 0 |
| C. Toss a coin to decide between option A and option B | 1.83% | 2 |
| Total Respondents: 109 | | |

## Q49 Read each item carefully before responding. Answer as honestly and accurately as you can. An answer of 1 (furthest to the left) means "Does not describe me well at all", and a response of 5 (furthest to the right) means "Describes me very well"

Answered: 109 Skipped: 13

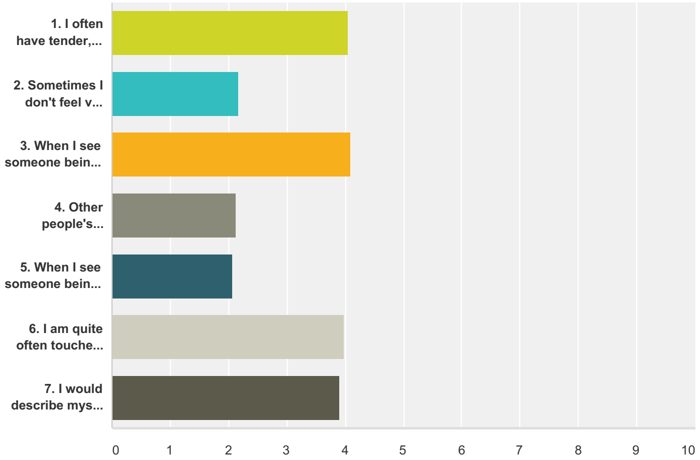


| | 1 | 2 | 3 | 4 | 5 | Total | Weighted Average |
|---|---|---|---|---|---|---|---|
| 1. I often have tender, concerned feelings for people less fortunate than me. | 1.83%<br>2 | 9.17%<br>10 | 11.01%<br>12 | 39.45%<br>43 | 38.53%<br>42 | 109 | 4.04 |

| | | | | | | | |
|---|---|---|---|---|---|---|---|
| 2. Sometimes I don't feel very sorry for other people when they are having problems. | 30.28% 33 | 42.20% 46 | 11.93% 13 | 11.93% 13 | 3.67% 4 | 109 | 2.17 |
| 3. When I see someone being taken advantage of, I feel kind of protective towards them. | 1.83% 2 | 5.50% 6 | 11.93% 13 | 43.12% 47 | 37.61% 41 | 109 | 4.09 |
| 4. Other people's misfortunes do not usually disturb me a great deal. | 32.11% 35 | 39.45% 43 | 14.68% 16 | 11.01% 12 | 2.75% 3 | 109 | 2.13 |
| 5. When I see someone being treated unfairly, I sometimes don't feel very much pity for them. | 35.78% 39 | 38.53% 42 | 11.93% 13 | 10.09% 11 | 3.67% 4 | 109 | 2.07 |
| 6. I am quite often touched by things that I see happen. | 2.75% 3 | 6.42% 7 | 14.68% 16 | 42.20% 46 | 33.94% 37 | 109 | 3.98 |
| 7. I would describe myself as a pretty soft-hearted person | 1.83% 2 | 8.26% 9 | 21.10% 23 | 34.86% 38 | 33.94% 37 | 109 | 3.91 |

## Q50 The following statements ask about your thoughts and feelings in a variety of situations. For each item, show how well it describes you by choosing the appropriate number on the scale from -4 to +4. + 4 = Very strong agreement+3 = Strong agreement+2 = Moderate agreement+1 = Slight agreement0 = Neither agreement nor disagreement-1 = Slight disagreement-2 = Moderate disagreement-3 = Strong disagreement-4 = Very strong disagreement

Answered: 109 Skipped: 13

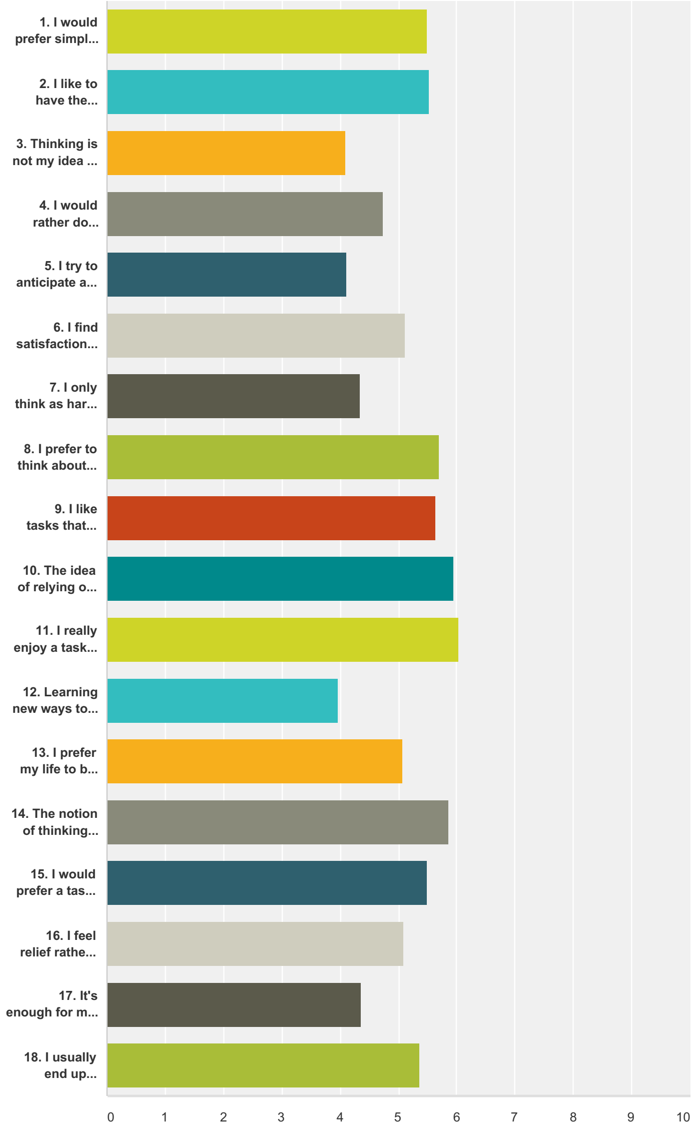


| | -4 | -3 | -2 | -1 | 0 | +1 | +2 | +3 | +4 | Total | Weighted Average |
|---|---|---|---|---|---|---|---|---|---|---|---|
| 1. I would prefer simple to complex problems. | 5.50% 6 | 6.42% 7 | 12.84% 14 | 12.84% 14 | 11.01% 12 | 11.01% 12 | 16.51% 18 | 10.09% 11 | 13.76% 15 | 109 | 5.50 |

| | | | | | | | | | | | |
|---|---|---|---|---|---|---|---|---|---|---|---|
| 2. I like to have the responsibility of handling a situation that requires a lot of thinking. | **5.50%**<br>6 | **6.42%**<br>7 | **11.01%**<br>12 | **7.34%**<br>8 | **11.93%**<br>13 | **14.68%**<br>16 | **22.02%**<br>24 | **19.27%**<br>21 | **1.83%**<br>2 | 109 | 5.53 |
| 3. Thinking is not my idea of fun. | **9.17%**<br>10 | **24.77%**<br>27 | **17.43%**<br>19 | **12.84%**<br>14 | **8.26%**<br>9 | **5.50%**<br>6 | **8.26%**<br>9 | **9.17%**<br>10 | **4.59%**<br>5 | 109 | 4.09 |
| 4. I would rather do something that requires little thought than something that is sure to challenge my thinking abilities. | **8.26%**<br>9 | **11.01%**<br>12 | **18.35%**<br>20 | **12.84%**<br>14 | **14.68%**<br>16 | **7.34%**<br>8 | **7.34%**<br>8 | **12.84%**<br>14 | **7.34%**<br>8 | 109 | 4.74 |
| 5. I try to anticipate and avoid situations where there is likely a chance I will have to think in depth about something. | **15.60%**<br>17 | **23.85%**<br>26 | **10.09%**<br>11 | **10.09%**<br>11 | **10.09%**<br>11 | **6.42%**<br>7 | **8.26%**<br>9 | **9.17%**<br>10 | **6.42%**<br>7 | 109 | 4.12 |
| 6. I find satisfaction in deliberating hard and for long hours. | **12.84%**<br>14 | **7.34%**<br>8 | **7.34%**<br>8 | **11.93%**<br>13 | **10.09%**<br>11 | **10.09%**<br>11 | **22.94%**<br>25 | **13.76%**<br>15 | **3.67%**<br>4 | 109 | 5.12 |
| 7. I only think as hard as I have to. | **14.68%**<br>16 | **17.43%**<br>19 | **11.93%**<br>13 | **11.93%**<br>13 | **4.59%**<br>5 | **16.51%**<br>18 | **9.17%**<br>10 | **8.26%**<br>9 | **5.50%**<br>6 | 109 | 4.35 |
| 8. I prefer to think about small, daily projects to long-term ones. | **4.59%**<br>5 | **3.67%**<br>4 | **12.84%**<br>14 | **6.42%**<br>7 | **18.35%**<br>20 | **13.76%**<br>15 | **15.60%**<br>17 | **13.76%**<br>15 | **11.01%**<br>12 | 109 | 5.69 |
| 9. I like tasks that require little thought once I've learned them. | **4.59%**<br>5 | **4.59%**<br>5 | **11.01%**<br>12 | **9.17%**<br>10 | **17.43%**<br>19 | **14.68%**<br>16 | **14.68%**<br>16 | **11.93%**<br>13 | **11.93%**<br>13 | 109 | 5.64 |
| 10. The idea of relying on thought to make my way to the top appeals to me. | **9.17%**<br>10 | **5.50%**<br>6 | **4.59%**<br>5 | **5.50%**<br>6 | **8.26%**<br>9 | **11.01%**<br>12 | **23.85%**<br>26 | **24.77%**<br>27 | **7.34%**<br>8 | 109 | 5.94 |
| 11. I really enjoy a task that involves coming up with new solutions to problems. | **7.34%**<br>8 | **6.42%**<br>7 | **7.34%**<br>8 | **2.75%**<br>3 | **8.26%**<br>9 | **11.93%**<br>13 | **20.18%**<br>22 | **25.69%**<br>28 | **10.09%**<br>11 | 109 | 6.04 |
| 12. Learning new ways to think doesn't excite me very much. | **18.35%**<br>20 | **21.10%**<br>23 | **14.68%**<br>16 | **11.01%**<br>12 | **2.75%**<br>3 | **7.34%**<br>8 | **13.76%**<br>15 | **5.50%**<br>6 | **5.50%**<br>6 | 109 | 3.96 |
| 13. I prefer my life to be filled with puzzles that I must solve. | **10.09%**<br>11 | **9.17%**<br>10 | **9.17%**<br>10 | **3.67%**<br>4 | **17.43%**<br>19 | **21.10%**<br>23 | **14.68%**<br>16 | **11.93%**<br>13 | **2.75%**<br>3 | 109 | 5.07 |
| 14. The notion of thinking abstractly is appealing to me. | **6.42%**<br>7 | **5.50%**<br>6 | **7.34%**<br>8 | **4.59%**<br>5 | **12.84%**<br>14 | **17.43%**<br>19 | **18.35%**<br>20 | **16.51%**<br>18 | **11.01%**<br>12 | 109 | 5.86 |
| 15. I would prefer a task that is intellectual, difficult, and important to one that is somewhat important but does not require much thought. | **9.17%**<br>10 | **8.26%**<br>9 | **7.34%**<br>8 | **5.50%**<br>6 | **11.93%**<br>13 | **12.84%**<br>14 | **21.10%**<br>23 | **19.27%**<br>21 | **4.59%**<br>5 | 109 | 5.50 |
| 16. I feel relief rather than satisfaction after completing a task that required a lot of mental effort. | **7.34%**<br>8 | **11.01%**<br>12 | **12.84%**<br>14 | **9.17%**<br>10 | **13.76%**<br>15 | **12.84%**<br>14 | **12.84%**<br>14 | **12.84%**<br>14 | **7.34%**<br>8 | 109 | 5.09 |
| 17. It's enough for me that something gets the job done; I don't care how or why it works. | **14.68%**<br>16 | **18.35%**<br>20 | **14.68%**<br>16 | **9.17%**<br>10 | **8.26%**<br>9 | **8.26%**<br>9 | **7.34%**<br>8 | **11.93%**<br>13 | **7.34%**<br>8 | 109 | 4.36 |
| 18. I usually end up deliberating about issues even when they do not affect me personally. | **6.42%**<br>7 | **11.93%**<br>13 | **10.09%**<br>11 | **7.34%**<br>8 | **8.26%**<br>9 | **19.27%**<br>21 | **17.43%**<br>19 | **6.42%**<br>7 | **12.84%**<br>14 | 109 | 5.36 |

## Q51 Please indicate the extent to which you feel positive or negative towards each of the following 12 issues by ranking each issue with a number from 0 to 100. Scores of 0 indicate greater negativity, and scores of 100 indicate greater positivity. A score of 50 indicates that you feel neutral about the issue.

Answered: 109 Skipped: 13

! We're sorry. We cannot display a chart for a question with this many options.

**Choose a number**

| | 1 | 2 | 3 | 4 | 5 | 6 | 7 | 8 | 9 | 10 | 11 |
|---|---|---|---|---|---|---|---|---|---|---|---|
| 1. Abortion | **20.18%**<br>22 | **0.92%**<br>1 | **1.83%**<br>2 | **0.00%**<br>0 | **1.83%**<br>2 | **0.92%**<br>1 | **0.00%**<br>0 | **0.92%**<br>1 | **0.00%**<br>0 | **4.59%**<br>5 | **0.92%**<br>1 |
| 2. Limited government | **2.75%**<br>3 | **0.92%**<br>1 | **0.00%**<br>0 | **0.92%**<br>1 | **0.92%**<br>1 | **0.00%**<br>0 | **0.92%**<br>1 | **0.00%**<br>0 | **0.00%**<br>0 | **6.42%**<br>7 | **0.00%**<br>0 |
| 3. Military and national security | **1.83%**<br>2 | **0.00%**<br>0 | **0.92%**<br>1 | **0.00%**<br>0 | **0.92%**<br>1 | **0.92%**<br>1 | **0.00%**<br>0 | **0.00%**<br>0 | **0.00%**<br>0 | **4.59%**<br>5 | **0.00%**<br>0 |

| | 12 | 13 | 14 | 15 | 16 | 17 | 18 | 19 | 20 | 21 | 22 |
|---|---|---|---|---|---|---|---|---|---|---|---|
| 1. Abortion | **0.00%**<br>0 | **0.00%**<br>0 | **0.00%**<br>0 | **0.00%**<br>0 | **0.00%**<br>0 | **0.00%**<br>0 | **0.00%**<br>0 | **0.00%**<br>0 | **2.75%**<br>3 | **0.00%**<br>0 | **0.00** [illegible] |
| 2. Limited government | **0.00%**<br>0 | **0.00%**<br>0 | **1.83%**<br>2 | **1.83%**<br>2 | **0.00%**<br>0 | **0.92%**<br>1 | **0.00%**<br>0 | **1.83%**<br>2 | **1.83%**<br>2 | **0.00%**<br>0 | **0.00** [illegible] |
| 3. Military and national security | **1.83%**<br>2 | **0.00%**<br>0 | **0.00%**<br>0 | **0.00%**<br>0 | **0.00%**<br>0 | **0.00%**<br>0 | **0.00%**<br>0 | **4.59%**<br>5 | **3.67%**<br>4 | **0.00%**<br>0 | **0.00** [illegible] |

| | | | | | | | | | | | | | | | | | | | | | | |
|---|---|---|---|---|---|---|---|---|---|---|---|---|---|---|---|---|---|---|---|---|---|---|
| 4. Religion | 19.27%<br>21 | 1.83%<br>2 | 0.00%<br>0 | 0.00%<br>0 | 4.59%<br>5 | 0.92%<br>1 | 0.00%<br>0 | 0.00%<br>0 | 0.00%<br>0 | 5.50%<br>6 | 0.92%<br>1 | 0.00%<br>0 | 0.92%<br>1 | 0.92%<br>1 | 1.83%<br>2 | 0.00%<br>0 | 0.00%<br>0 | 0.00%<br>0 | 0.00%<br>0 | 0.92%<br>1 | 0.00%<br>0 | 0.00 |
| 5. Welfare benefits | 1.83%<br>2 | 0.92%<br>1 | 0.00%<br>0 | 0.92%<br>1 | 0.00%<br>0 | 0.92%<br>1 | 0.00%<br>0 | 0.00%<br>0 | 0.92%<br>1 | 3.67%<br>4 | 0.92%<br>1 | 0.00%<br>0 | 0.00%<br>0 | 0.92%<br>1 | 0.92%<br>1 | 0.00%<br>0 | 0.92%<br>1 | 0.00%<br>0 | 0.92%<br>1 | 3.67%<br>4 | 0.00%<br>0 | 0.00 |
| 6. Gun ownership | 14.68%<br>16 | 0.92%<br>1 | 0.00%<br>0 | 0.92%<br>1 | 3.67%<br>4 | 1.83%<br>2 | 0.00%<br>0 | 0.00%<br>0 | 0.00%<br>0 | 5.50%<br>6 | 0.00%<br>0 | 0.00%<br>0 | 0.92%<br>1 | 0.92%<br>1 | 1.83%<br>2 | 1.83%<br>2 | 1.83%<br>2 | 0.00%<br>0 | 0.92%<br>1 | 7.34%<br>8 | 0.00%<br>0 | 0.00 |
| 7. Traditional marriage | 13.76%<br>15 | 0.92%<br>1 | 0.00%<br>0 | 0.92%<br>1 | 1.83%<br>2 | 1.83%<br>2 | 0.92%<br>1 | 0.00%<br>0 | 0.00%<br>0 | 2.75%<br>3 | 0.00%<br>0 | 0.00%<br>0 | 0.00%<br>0 | 0.00%<br>0 | 0.92%<br>1 | 0.00%<br>0 | 0.00%<br>0 | 0.92%<br>1 | 0.00%<br>0 | 0.92%<br>1 | 0.00%<br>0 | 0.00 |
| 8. Traditional values | 8.26%<br>9 | 1.83%<br>2 | 0.00%<br>0 | 0.00%<br>0 | 1.83%<br>2 | 0.00%<br>0 | 0.92%<br>1 | 0.00%<br>0 | 0.92%<br>1 | 5.50%<br>6 | 0.00%<br>0 | 0.00%<br>0 | 0.92%<br>1 | 0.00%<br>0 | 1.83%<br>2 | 0.92%<br>1 | 0.00%<br>0 | 0.00%<br>0 | 0.00%<br>0 | 0.92%<br>1 | 0.00%<br>0 | 0.00 |
| 9. Fiscal responsibility | 0.00%<br>0 | 0.00%<br>0 | 0.00%<br>0 | 0.00%<br>0 | 1.83%<br>2 | 0.00%<br>0 | 0.00%<br>0 | 0.92%<br>1 | 0.00%<br>0 | 1.83%<br>2 | 0.00%<br>0 | 0.00%<br>0 | 0.92%<br>1 | 0.00%<br>0 | 0.92%<br>1 | 0.00%<br>0 | 0.92%<br>1 | 0.00%<br>0 | 0.00%<br>0 | 0.00%<br>0 | 0.00%<br>0 | 0.00 |
| 10. Business | 0.92%<br>1 | 0.00%<br>0 | 0.92%<br>1 | 0.00%<br>0 | 1.83%<br>2 | 0.00%<br>0 | 0.00%<br>0 | 0.00%<br>0 | 0.92%<br>1 | 5.50%<br>6 | 0.00%<br>0 | 0.00%<br>0 | 0.92%<br>1 | 0.92%<br>1 | 1.83%<br>2 | 0.00%<br>0 | 0.00%<br>0 | 1.83%<br>2 | 0.00%<br>0 | 0.92%<br>1 | 0.00%<br>0 | 0.00 |
| 11. The family unit | 0.00%<br>0 | 0.00%<br>0 | 0.92%<br>1 | 0.00%<br>0 | 0.92%<br>1 | 0.00%<br>0 | 0.00%<br>0 | 0.00%<br>0 | 0.00%<br>0 | 0.00%<br>0 | 0.00%<br>0 | 0.00%<br>0 | 0.92%<br>1 | 0.92%<br>1 | 0.92%<br>1 | 0.00%<br>0 | 0.00%<br>0 | 0.00%<br>0 | 0.00%<br>0 | 0.00%<br>0 | 0.92%<br>1 | 0.00 |
| 12. Patriotism | 3.67%<br>4 | 0.00%<br>0 | 0.92%<br>1 | 0.00%<br>0 | 0.00%<br>0 | 0.92%<br>1 | 0.92%<br>1 | 0.92%<br>1 | 0.00%<br>0 | 4.59%<br>5 | 0.00%<br>0 | 0.00%<br>0 | 0.00%<br>0 | 0.00%<br>0 | 0.92%<br>1 | 0.92%<br>1 | 0.00%<br>0 | 0.00%<br>0 | 1.83%<br>2 | 1.83%<br>2 | 0.00%<br>0 | 0.00 |
